# Significant transcriptional changes in 15q duplication but not Angelman syndrome deletion stem cell-derived neurons

**DOI:** 10.1186/s13229-018-0191-y

**Published:** 2018-01-27

**Authors:** Nora Urraca, Kevin Hope, A. Kaitlyn Victor, T. Grant Belgard, Rawaha Memon, Sarita Goorha, Colleen Valdez, Quynh T. Tran, Silvia Sanchez, Juanma Ramirez, Martin Donaldson, Dave Bridges, Lawrence T. Reiter

**Affiliations:** 1 Department of Neurology, The University of Tennessee Health Science Center, 855 Monroe Ave., Link 415, Memphis, TN 38163 USA; 2 IPBS Program, The University of Tennessee Health Science Center, Memphis, TN 38163 USA; 3 Department of Pediatrics, The University of Tennessee Health Science Center, Memphis, TN 38163 USA; 4 Department of Pediatric Dentistry, The University of Tennessee Health Science Center, Memphis, TN 38163 USA; 5 Department of Preventive Medicine, The University of Tennessee Health Science Center, Memphis, TN 38163 USA; 6 Department of Nutritional Sciences, University of Michigan School of Public Health, 1415 Washington Heights, Ann Arbor, MI 48109 USA; 7 Instituto Nacional de Pediatria, 04530 Mexico City, Mexico; 8 Department of Biochemistry and Molecular Biology, University of Basque Country, Bilbao, Spain; 9 MRC Functional Genomics Unit, Department of Physiology, Anatomy and Genetics, University of Oxford, Oxford, OX1 3QX UK

**Keywords:** Stem cells, Autism, Genomic disorders, Neurogenetic syndrome, mRNAseq

## Abstract

**Background:**

The inability to analyze gene expression in living neurons from Angelman (AS) and Duplication 15q (Dup15q) syndrome subjects has limited our understanding of these disorders at the molecular level.

**Method:**

Here, we use dental pulp stem cells (DPSC) from AS deletion, 15q Duplication, and neurotypical control subjects for whole transcriptome analysis. We identified 20 genes unique to AS neurons, 120 genes unique to 15q duplication, and 3 shared transcripts that were differentially expressed in DPSC neurons vs controls.

**Results:**

Copy number correlated with gene expression for most genes across the 15q11.2-q13.1 critical region. Two thirds of the genes differentially expressed in 15q duplication neurons were downregulated compared to controls including several transcription factors, while in AS differential expression was restricted primarily to the 15q region. Here, we show significant downregulation of the transcription factors *FOXO1* and *HAND2* in neurons from 15q duplication, but not AS deletion subjects suggesting that disruptions in transcriptional regulation may be a driving factor in the autism phenotype in Dup15q syndrome. Downstream analysis revealed downregulation of the ASD associated genes *EHPB2* and *RORA*, both genes with FOXO1 binding sites. Genes upregulated in either Dup15q cortex or idiopathic ASD cortex both overlapped significantly with the most upregulated genes in Dup15q DPSC-derived neurons.

**Conclusions:**

Finding a significant increase in both *HERC2* and *UBE3A* in Dup15q neurons and significant decrease in these two genes in AS deletion neurons may explain differences between AS deletion class and *UBE3A* specific classes of AS mutation where *HERC2* is expressed at normal levels. Also, we identified an enrichment for FOXO1-regulated transcripts in Dup15q neurons including ASD-associated genes *EHPB2* and *RORA* indicating a possible connection between this syndromic form of ASD and idiopathic cases.

**Electronic supplementary material:**

The online version of this article (10.1186/s13229-018-0191-y) contains supplementary material, which is available to authorized users.

## Background

The study of neurogenetic syndromes in the laboratory setting is inherently complicated by a lack of easy access to living neurons for gene expression and electrophysiological studies. Although a variety of stem cell types has been used in the laboratory, including embryonic stem cells, blood stem cells, and most notably induced pluripotent stem cells (iPSCs) derived from skin biopsies [1], there are still technical issues complicating the use of these limited resources. As an alternative to iPSC, dental pulp stem cells (DPSC) are neural crest-derived multipotent cells that can be easily obtained from deep within the pulp of shed (deciduous) teeth that either fall out on their own or are extracted in the pediatric clinic setting when ready for exfoliation. DPSC have been differentiated into a variety of cell types including osteoblasts, hepatocytes, insulin-producing cells, and even neurons [2]. Furthermore, neurons derived from DPSC display action potentials (Na^+^ currents) [3, 4] and are positive for neuronal markers like MAP2 and TUJ1 [5]. In our hands, DPSC-derived neurons have not been able to survive in vitro long enough to become mature neurons unless they are first immortalized. We are confident, however, that these immature neuronal cultures are an excellent resource for gene expression studies in the disease state, especially for neurodevelopmental disorders where the phenotypes appear at or near birth. In addition, we recently showed that DPSC maintain an epigenetic landscape more similar to embryonic stem cells than iPSC making them ideal for gene expression studies [6]. DPSC can also be easily made into neurons without the use of viral vectors. Finally, the collection of DPSC from individuals with these syndromes is quite easy and non-invasive. Sample collection can even take place through shipments of exfoliated teeth by parents from remote locations where the subjects reside.

Recently, our laboratory performed an extensive molecular characterization of both DPSC and DPSC-derived neuronal cultures [5]. We established that the process of differentiation from DPSC to neurons is primarily driven by the downregulation of the REST transcriptional repressor, turning on a number of genes involved in neuronal differentiation and function producing cells that show a strong induction of the neuron-specific MAP2 marker and residual expression of glial marker GFAP indicating that these cultures are representative of the mixed neuron/glia characteristics of the human brain [5].

**Fig. 1:**
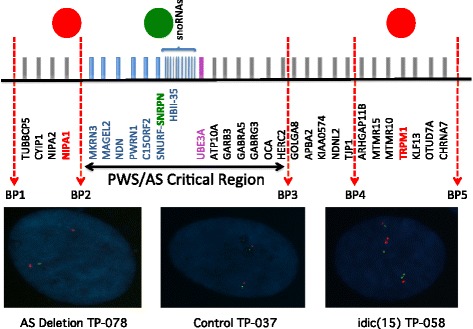
FISH confirmation of duplication/deletion boundaries. The colored circles on the top of the figure represent FISH probes for *NIPA1* (red), *SNRPN* (green), and *TRPM1* (red). These probes were used to determine the duplication/deletion status for all cell lines and to establish if these duplications/deletions included BP1-BP2 and or BP4-BP5 genes. Signals for AS deletion TP-078 (*NIPA1* and *SNRPN*) indicate a deletion only of the BP2-BP3 region on one allele; signals for the control TP-037 line (*NIPA1* and *SNRPN*) indicate two copies of the BP1-BP3 region; and signals for idic(15) TP-058 (*TRPM1* and *SNRPN*) indicate the presence of four copies of BP2-BP3 as well as BP4-BP5

Here, we provide compelling evidence that DPSC neuronal cultures can be used for the molecular investigation of neurons in the disease state by analyzing gene expression in DPSC neuronal cultures from two distinct, but molecularly related, syndromes. We focused this study on two reciprocal genomic disorders: duplication of proximal 15q11.2-q13.1 (Dup 15q syndrome MIM: 608636) resulting in one of the most common forms of autism with an identifiable molecular lesion [7–10] and Angelman syndrome (AS MIM: 105830) which is caused, in ~ 75–80% of cases [11], by a deletion of this same critical region of ~ 5Mb on chromosome 15q11.2-q13.1 (Fig. 1).

While it is apparent that the key components of the Angelman syndrome phenotype are caused by loss of function of the maternally expressed *UBE3A* gene in the brain [12, 13], it is still unclear how loss of *UBE3A* can directly lead to the observed neurological defects including synaptic plasticity problems, ataxia, seizures, and intellectual disability [14]. Significant effort has surrounded the search for direct targets of the UBE3A ubiquitin ligase in model systems from flies to mice [15–20]. However, little progress has been made in identifying a single substrate or even pathway that can explain the plasticity, motor, and learning/memory deficits in these animal models. One aspect of UBE3A function that has largely been ignored is the observation that UBE3A can act as a transcriptional regulator in addition to its role as a ubiquitin ligase [21, 22].

Almost nothing is known about the underlying pathogenesis of Duplication 15q syndrome, caused by interstitial and isodicentric duplications of 15q11.2-q13.1 that may involve several genes in the duplicated region to produce the autism spectrum disorder (ASD) and seizure phenotypes found in patients with this disorder [7, 10, 23]. Some studies have indicated that the maternally expressed *UBE3A* gene may be a major contributor to the autism phenotype in these individuals since in both interstitial and isodicentric duplications, there is a clear bias towards maternally derived or inherited duplications where ASD is present [24–26]. Some evidence also suggests that the seizure phenotype in these individuals may be related to over-expression of a GABA (A) receptor subunit gene cluster also located in the critical BP2-BP3 duplicated region [7]. However, it has been difficult to definitively establish that the GABA receptor genes are indeed elevated in 15q Duplication post-mortem brain tissue [27, 28] or blood [29]. The contribution of other genes, both within the duplicated region and elsewhere in the genome, to the 15q Duplication syndrome phenotype is almost entirely speculative at this time since no comprehensive analysis of gene expression from Duplication 15q patient neurons is currently available.

In this study, we interrogated genome-wide gene expression changes in DPSC and DPSC neuronal cultures from individuals with both AS deletions and 15q duplications in an effort to establish which gene networks may be involved in the phenotypes of these two syndromes, but also to directly compare the consequences on gene expression for duplication/deletion of the same chromosomal region that ties these two disorders at the genomic level. Whole genome gene expression analysis of patient-derived DPSC neuronal cultures from these disorders has revealed several key regulators of gene expression and neurologically relevant genes which may substantially contribute to the 15q duplication syndrome phenotype, while the number of gene expression changes in the Angelman syndrome set remained quite small. Perhaps most surprising was the extensive change observed for transcription factor encoded genes in the 15q Duplication syndrome subjects, suggesting global transcriptional changes mediated by transcription factors FOXO1 and/or HAND2 may be driving the pathogenesis of ASD in this syndrome.

## Methods

### Generation of dental pulp stem cell cultures

Control teeth were obtained through the Department of Pediatric Dentistry at the University of Tennessee Health Science Center (UTHSC). Teeth from AS deletion and Dup15q subjects were shipped from various locations by the parents of children with these conditions. The UTHSC Institutional Review Board (IRB) approved this study, and informed consent was obtained in accordance with IRB policy. Immediately following extraction of a loose tooth in the clinic, it was placed in transportation media (DMEM/F12 50/50 mix with HEPES (Fisher Scientific, Waltham, MA) and 100 U/mL penicillin, 100 μg/mL streptomycin). DPSC were isolated and cultured as previously described with slight modifications [30]. Briefly, the pulp was minced and digested in a solution of 3 mg/mL Collagenase type I and 4 mg/mL Dispase II for 1 h at 37 °C. Cells were seeded in poly-D-Lysine 12-well dishes and maintained under standard conditions (37 °C, 5% CO_2_) in DMEM/F12 1:1, 10% fetal bovine serum (FBS) (Fisher Scientific, Waltham, MA), 10% newborn calf serum (NCS) (Fisher Scientific, Waltham, MA) and 100 U/mL penicillin, and 100 μg/mL streptomycin (Pen/Strep). Sub-confluent cultures were passaged regularly with 0.1 μM HyQTase (HyClone).

### Fluorescent in situ hybridization breakpoint analysis

Fluorescent in situ hybridization was used to determine the approximate breakpoints for either deletion or duplication DPSC lines. We used the pepsin method of 0.1 mg pepsin (Sigma-Aldrich, St. Louis, MO) in 40 ml of 0.01N HCl in order to permeabilize the nuclear membrane for access to the DNA. Three different probes localized in chromosome 15q were used: *NIPA1* (BP1-BP2) and *TRPM1* (BP4-BP5) in the red spectrum and *SNRPN* (BP2-BP3) in the green spectrum (SURE FISH, Agilent, Foster City, CA). Hybridization was carried out at RT overnight. Fluorescent signal analysis was performed according to published protocols [31] using a Leica DM6000 upright fluorescent microscope running the Leica application Suite Imaging software (Leica Microsystems, Wetzlar, Germany). At least 15 preparations were counted per cell line.

### Neural differentiation

Early passage (2–3) primary DPSC were converted to neurons according to a previously published protocol [30]. 20,000 cells/cm^2^ were seeded in poly-D-lysine-coated 12-well plates or T-25 flasks in DMEM/F12 (1:1), 2.5% FCS, 100 U/mL penicillin, and 100 μg/mL streptomycin, and cultured for 24 h. Epigenetic reprogramming was performed by exposing the DPSC to 10 μM 5-azacytidine (Acros Scientific, Geel, Belgium) in DMEM/F12 containing 2.5% FCS (Fisher Scientific, Waltham, MA) and 10 ng/mL bFGF for 48 h. Neural differentiation was induced by exposing the cells to 250 μM IBMX, 50 μM forskolin, 200 nM TPA (Sigma Aldrich, St. Louis, MO), 1 mM db-cAMP, 10 ng/mL bFGF, 10 ng/mL NGF (Invitrogen, Carlsbad, CA), 30 ng/mL NT-3 (Peprotech, Rocky Hill, NJ), and 1% insulin-transferrin-sodium selenite premix (ITS) in DMEM/F12 for 3 days. At the end of the neural induction treatment, the cells were washed with 1X PBS. Neuronal maturation was performed by maintaining the cells in Neurobasal A media (Invitrogen, Carlsbad, CA) supplemented with 1 mM dbcAMP, 1% N2, 1% B27, and 30 ng/mL NT-3 and 1X Glutamax for 3 weeks.

### RNA sequencing of DPSC and DPSC-neurons

Total RNA was extracted from ~ 3-week cultured neurons and DPSC using the miRNeasy Mini kit from Qiagen (Foster City, CA). Total RNA was analyzed on an Agilent Bioanalyzer 6000 pico chip and determined to have a RIN of > 8.0. 5 ng of total RNA and was then used to prepare dscDNA using the NuGEN Ovation V2 kit. Five hundred nanograms of this dscDNA then used to prepare libraries for sequencing using the AB Library Builder™ Fragment library Kit for 5500 Genetic Analysis Systems on a Library Builder system. Libraries were amplified 7 cycles before 5500 Wildfire primers added by 5 cycles of fusion primer amplification as described in the 5500 Wildfire manual (Applied Biosystems, Foster City, CA). Before sequencing on the ABI system, small aliquots of this material pooled and sequenced on an Ion Torrent PGM 314 chip after additional amplification with PGM fusion primers to estimate sample abundance. Barcode quantification data from the PGM was used to pool the samples with Wildfire primers equally. Following this final pooling, the library pools were examined and quantified on an Agilent high sensitivity DNA chip, immobilized on flow cells for the 5500 Wildfire instrument, and sequenced (50 bp reads) on this instrument. On average, a total of 30 million reads were produced per sample for downstream analysis. Complete sequencing reads for this study are available on the Gene Expression Omnibus (GEO) website using query [GEO: GSE67122].

### Bioinformatic analysis

ABI files were converted from XSQ format to separate quality and colorspace fasta files for analysis using the TopHat with Bowtie pipeline [32]. Short transcript fragments were mapped to the reference human genome (GRCh38; accession number GRCh38.77) using TopHat v2.0.13 [33] with Bowtie v2.1.0 [34] and Samtools v0.1.19. Mapped reads were indexed to known genes using HTseq v0.6.1 [35]. Differentially expressed transcripts were identified using the DESeq2 bioconductor package [36]. Heatmaps were generated using the R software package (v 3.1.1) [37]. Venn diagrams were generated online using Gene List Venn Diagram software, additional descriptive analysis was performed using Gene Set Enrichment Analysis (GSEA) using a rank-ordered gene list based on fold change that was used to identify pathways and transcription factor binding motifs enriched in particular data sets [38]. Statistical significance for GSEA analysis was set at *q* ≤ 0.25 as per the developer’s suggestion. Gene sets were obtained from MSigDB version 4.0. Gene sets that were found to be significantly up- or downregulated (*q* ≤ 0.05) were also analyzed as a gene list with default stringency settings using The **D**atabase for **A**nnotation, **V**isualization and **I**ntegrated **D**iscovery (DAVID v6.7).

### Quantitative real-time PCR analysis

Total RNA was extracted from differentiated neurons using a Direct-zol RNA miniprep plus kit (Zymogen R0270) according to the manufacturer’s instructions and quantified by spectrophotometry (NanoDrop Technologies). Five hundred nanograms of total RNA was used as input for each cDNA synthesis reaction using the Transcriptor First Strand cDNA Synthesis Kit (Roche, 04379012001). cDNA reactions were diluted 1:10 and three technical replicates were performed on each sample for each gene tested using the default cycling parameters of the Light Cycler 480 system (Roche). For primers and probes, see Additional file 1: Table S9. The crossing point (Cp) value was calculated using the absolute quantitation algorithm (Roche) for each sample. Delta Cp was calculated by subtracting the *GAPDH* control gene Cp from the Cp of the test gene. Statistical significance was determined by unpaired *t* tests corrected for multiple comparisons.

### Quantitative western blot analysis

Infrared (IR) Western blots and quantification from DPSC neurons were performed as previously described in Urraca et al. [5] using six unrelated DPSC lines from neurotypical controls and idic15q. These six independent DPSC lines were not run for RNAseq analysis as they were collected after the RNAseq runs. We used this sample to validate the RNAseq findings in an independent cohort. The α-RORA antibody (Aviva Biosystems # OAAN02076) was used at a dilution of 1:500 in conjunction with Li-Cor α-Rabbit 800 secondary antibody at 1:2500. Loading control was α-GAPDH with α-Goat 680 at 1:2500. Each individual sample was normalized to the GAPDH signal as a loading control, and quantification was performed using all 12 samples in three independent Western blots by Student’s *t* test (control vs idic15).

For the analysis of neural differentiation markers, we used three control and three idic15 lines per group. All primary antibodies used were from AbCam (Cambridge, MA). Primary concentrations were 1:1000 for GABAA (ab98968), NLG1 (ab26305), PSD95 (ab12093), and UBE3A (ab126765) and 1:2000 for MAP2 (ab5392) and GAPDH (ab157156). Secondary IR antibodies and quantification were performed as above for RORA analysis.

### Comparison to postmortem Dup15q and ASD brain tissue

The most significant differentially expressed genes between control and Dup15q DPSC neurons were directly compared to a combined list of temporal and frontal cortical gene expression from a study using postmortem brain from Dup15q and ASD individuals (see [39]). This analysis was also repeated after removing any bias from genes in the 15q11.2-q13.1 duplicated region. We performed PANTHER over-representation tests (release 20150430) on the significantly overlapping intersections between the 1500 genes that went up most significantly in DPSC neurons in Dup15q vs controls and the genes that went up significantly in postmortem Dup15q cortex or postmortem idiopathic ASD cortex at an FDR of 5% [39]. These comparisons were performed using default settings for every class of annotations, filtering only those terms significant at a Bonferroni-corrected *p* ≤ 0.05 for either of the two overlaps. The background was the intersection of genes expressed in Parikshak et al. and genes assigned a *p* value by DESeq in the current study.

## Results

### Identification of breakpoints in DPSC lines

Teeth used for this study were primarily obtained long distance using a biospecimen tube filled with cell culture media and a pre-addressed return package. As such, in most cases, we only knew that an individual has a diagnosis of Angelman syndrome or 15q Duplication syndrome from the genetics report, which often did not provide detailed information about critical breakpoints for duplication/deletion in this region. The critical region for both AS and 15q Duplication syndrome contains ~ 15 protein coding genes and is located between BP2 and BP3 in Fig. 1. We performed fluorescent in situ hybridization (FISH) analysis on DPSC cell lines from all subjects whose samples were used for mRNA-seq analysis in order to determine if the AS deletion samples included the BP1-BP2 region and to determine if the 15q duplication samples were duplicated for the BP4-BP5 region. FISH signals indicated that all AS deletion samples are deleted for the region between BP2 and BP3 on the maternal chromosome (Table 1). The isodicentric Duplication 15q samples showed signals indicative of inclusion of the BP4 to BP5 region in all cases resulting in four copies of the entire region from BP5 to the centromere in these samples (Table 1). FISH analysis of copy number across the 15q region for all samples used in the study is in Additional file 2: Fig. S1.

**Table 1:** FISH results to determine breakpoints in DPSC lines

Cell line	Disease state	FISH PROBE		
		NIPA1 BP1-BP2	SNRPN BP2-BP3	TRPM1 BP4-BP5
TP-023	Not affected	2 copies	2 copies	2 copies
TP-037	Not affected	2 copies	2 copies	2 copies
TP-076	Not affected	2 copies	2 copies	2 copies
TP-055	AS	1 copy	1 copy	2 copies
TP-059	AS	2 copies	1 copy	2 copies
TP-078	AS	2 copies	1 copy	2 copies
TP-041	Interstitial Dup15q	2 copies	3 copies	2 copies
TP-044	idic(15)	4 copies	4 copies	3 copies
TP-058	idic(15)	4 copies	4 copies	4 copies

All Control DPSC showed the normal number of signals for each probe indicating no duplications or deletions in the region between BP1-BP5 in Fig. 1. AS sample TP-055 did not show a signal for probe NIPA1 indicating that this deletion is a BP1-BP3 deletion. AS samples TP-059 and TP-078 appear to be BP2-BP3 deletions. Sample TP-041 appears to be a BP2-BP3 interstitial duplication of 15q, while samples TP-044 and TP-058 are isodicentric duplications that include four copies of the entire BP1-BP5 region. See Additional file 2: Fig. S1 for example FISH images.

### mRNA-seq analysis of the 15q duplication/deletion critical region

Whole genome mRNA sequencing (mRNA-seq) was performed on nine subjects for both DPSC and DPSC neuronal cultures with a total of 18 samples (DPSC: 3 control, 3 AS deletion, 3 15q duplication and DPSC-neurons: 3 control, 3 AS deletion, 3 15q duplication). DPSC lines were differentiated into neurons using previously published protocols [30], and the DPSC neuronal cultures used for mRNA-seq studies were allowed to mature for at least 3 weeks prior to RNA extraction. Previous studies indicate that longer neuronal maturation times (up to 10 weeks) result in significant cell death and consequently poor RNA yield and that massive gene expression changes have already occurred in these cultures by ~ 3 weeks including a switch from MAP2 negative to MAP2 positive status [5]. Based on these studies and the potential high RNA yield, we chose to use ≥ 3-week old DPSC neuron cultures for the present study. Tooth samples consisted of three neurotypical controls obtained locally, three AS deletion cases, and three 15q duplication cases (one interstitial BP2-BP3 duplication and two idic(15) cases which included BP4-BP5) collected by the parent or guardian and shipped to the laboratory.

**Fig. 2:**
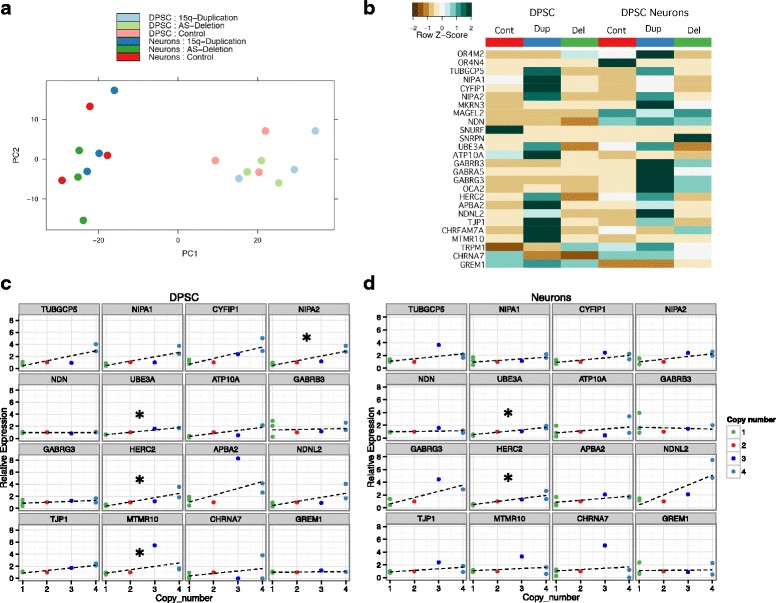
Gene expression changes across the 15q region. **a** principle component analysis. Note that the main differentiating feature of the gene expression data is the cell type (i.e., DPSC or DPSC-neuron) and not the genotype of the line. **b** Heatmap of gene expression for genes between BP1-BP3 on 15q11.2-q13.1. Note that some, but not all, genes show elevated expression in the DPSC and DPSC neuronal cultures from duplication lines. The expression differences are less striking for AS deletion lines where only a few specific genes appear significantly downregulated in DPSC and fewer still in the DPSC-neurons. **c** Graphs of copy number vs gene expression for DPSC in BP2-BP3 protein coding genes. Green dots are AS deletion, red dots are typical control, blue dot is interstitial Dup15q, and light blue dots are idic(15). Note that only *NIPA2*, *UBE3A*, *HERC2*, and *MTMR10* showed a significant correlation between copy number and gene expression. Star indicates that these genes showed a significant trend using Jonckheere’s trend test. **d** Graphs of copy number vs gene expression in neurons for the BP2-BP3 region. Note that only *UBE3A* and *HERC2* still show a significant trend test

In order to determine if genotype alone could explain the variance among the cell lines, we performed principle component analysis (PCA) across all samples using Reads Per Kilobase of exon per Million fragments (RPKM) mapped values as a measure of gene expression (Fig. 2a). We found two prominent groups separated, not by genotype, but by differentiation state (DPSC vs DPSC-derived neurons). The undifferentiated DPSC did not further stratify by disease state. The DPSC-derived neurons did show some separation by gene expression with the AS deletion samples showing somewhat lower global expression, but this observation did not reach significance. This analysis indicates that all samples had similar overall gene expression profiles regardless of genotype but clearly dependent on differentiation state from DPSC to neuronal cultures.

Next, we looked carefully at gene expression for genes located within the duplicated or deleted region as compared to neurotypical controls. A heatmap of the average gene expression across the three lines per group for protein coding genes across the proximal 15q region from the centromere to BP5 revealed that gene expression is elevated at multiple loci in the Dup15q samples as compared to neurotypical controls in both DPSC and DPSC-derived neurons (Fig. 2b). The AS deletion lines did not differ significantly in gene expression as compared to neurotypical controls for either DPSC or DPSC-derived neurons with a few notable exceptions: *UBE3A* expression levels followed copy number in both DPSC and neurons going up in 15q duplication and down in AS deletion; the *GABA A receptor* gene cluster consisting of *GABRB3*, *GABRG3*, and *GABRA5* each showed significantly elevated gene expression in 15q duplication neural cultures but were also slightly elevated in AS deletion samples; and the *HERC2* gene located at the distal breakpoint for the AS deletion (BP3) also showed significantly elevated gene expression in 15q duplication neural cultures and was significantly decreased in AS deletion cultures (Fig. 2c).

Next, we mapped normalized gene expression (normalized to controls) vs copy number for genes in the 15q region. Jonckheere’s trend tests were used to test the hypothesis that the median gene expression of all genes in the 15q region increases significantly as the copy number increases. Both DPSC and DPSC-derived neurons showed a significant trend with respect to copy number (*p* value ≤ 1 × 10^−4^) indicating that as copy number increased, so did gene expression for genes in the 15q region. Several genes followed this trend of copy number vs gene expression within the BP2-BP3 region shared by both the AS deletion and 15q duplication samples in both DPSC and DPSC-derived neurons (Fig. 2d). These genes included *UBE3A* and *HERC2* which were both significantly trending upward with copy number in both DPSC and DPSC-derived neurons (Table 2). Genes located just outside of the PWS/AS critical region but near the flanking breakpoints also showed a correlation between copy number and gene expression, but only for the 15q duplication samples since these AS deletion lines were only deleted between BP2-BP3 and therefore have 2X copies of the regions outside of the BP2-BP3. On the proximal side of the BP2-BP3 region, the *CYFIP1*, *NIPA1*, *NIPA2*, and *TUBGC5* genes all showed elevated expression in 15q duplication samples consistent with increased copy number (4X) for these genes in 15q duplication samples. On the distal side of the PWS/AS, there was a notable change in *CHNRA7* gene expression from inverse correlation in DPSC to correlated copy number and gene expression in DPSC neuronal cultures (Fig. 2d). Overall there was a positive correlation between DNA copy number and gene expression in the 15q region in both DPSC and DPSC-derived neurons.

**Table 2:** Jonckheere trend test for correlation between copy number and gene expression

Genes	DPSC		Neurons	
	*p* value	adj. *p*	*p* value	adj. *p*
TUBGCP5	0.057	0.105	0.140	0.224
NIPA1	0.021	0.066	0.139	0.224
CYFIP1	0.065	0.105	0.033	0.172
NIPA2	0.005	0.021	0.058	0.172
NDN	0.582	0.582	0.580	0.623
UBE3A	4.00E-04	0.006	0.002	0.014
ATP10A	0.194	0.239	0.495	0.609
GABRB3	0.336	0.384	0.139	0.224
GABRG3	0.060	0.105	0.195	0.284
HERC2	0.004	0.021	0.001	0.014
APBA2	0.094	0.126	0.059	0.172
NDNL2	0.062	0.105	0.065	0.172
TJP1	0.093	0.126	0.096	0.220
MTMR10	0.004	0.021	0.338	0.450
CHRNA7	0.481	0.513	0.584	0.623
GREM1	0.036	0.096	0.745	0.745

### Gene expression in AS and 15q duplication compared to controls

Global protein coding mRNA gene expression was compared between AS deletion and typical controls as well as 15q duplication and neurotypical controls for both DPSC and DPSC-derived neurons using DEseq to assess fold change and significantly differential whole transcript level expression [40]. Only 13 genes were found to be significantly different in AS deletion subjects and 7 genes in 15q duplication vs controls for the undifferentiated DPSC at an adjusted *p* value ≤ 0.05. None of these 20 genes was shared between the two disease states. After differentiation into neurons, 123 genes were significantly different (adj. *p* value ≤ 0.05) in the 15q duplication samples vs control and 23 genes in the AS deletion samples (adj. *p* value ≤ 0.05). Two of these genes, *DPT* and *AKRIC1*, were elevated in both 15q duplication and AS deletion samples (*F*_c_ = + 2.8) while *MED12L* gene expression decreased (*F*_c_ = − 2.8) in both groups. None of these genes was located in the PWS/AS critical region. Boxplots of all significantly different genes discussed in the text that are not located in the 15q11.2-q13.1 critical region can be found in Additional file 2: Fig. S2. However, when gene expression was compared between 15q duplication and AS deletion samples (both DPSC and DPSC-derived neurons), there were significant differences in gene expression for several genes in the 15q region. In DPSC *NIPA2*, *UBE3A*, *HERC2*, and *ATP10A*, all showed significant increase in gene expression (adj. *p* value ≤ 0.05) in 15q duplication cells vs AS deletion cells with a fold change ranging from 2.3–3.7. In DPSC-derived neurons, six genes (*NIPA2*, *UBE3A*, *HERC2*, *NDNL2*, *CYFIP1*, *TUBGCP5*) showed significantly higher expression in 15q duplication neurons (adj. *p* value ≤ 0.05) with a fold change ranging from 2.3–3.7 depending on the gene. Perhaps, the most interesting genes differentially regulated in the 15q duplication neurons were the *CCL7* gene, a chemokine associated with autoimmune response (adj. *p* value = 2 × 10^−3^; logF_c_ = + 2.0), and the two transcription factor genes *FOXO1*, also known as *FOXO1A* (adj-p_value_ = 3X10^−3^; *F*_c_ = − 3.4), a transcription factor that functions in the AKT signaling cascade and *HAND2* (adj. *p* value = 2.4 × 10^−5^; *F*_c_ = − 6.5). *HAND2* plays a role in the development of the sympathetic nervous system and could be involved in several Dup15q phenotypes [41–43]. In the AS deletion neurons, the most interesting single genes that changed were *SHISH2* (adj. *p* value = 3.5 × 10^−6^; *F*_c_ = − 4.9), an antagonist to both WNT and FGF signaling pathways, and *TRHDE* (adj. *p* value = 3 × 10^−4^; *F*_c_ = + 5.7) an extracellular peptidase that cleaves neuropeptide thyrotropin-releasing hormone and *CRY2* (adj. *p* value = 2 × 10^−2^; *F*_c_ = − 2.0). *CRY2* is a circadian clock gene, and circadian rhythm defects have been found in both fly and mouse models of AS [44, 45].

**Fig. 3:**
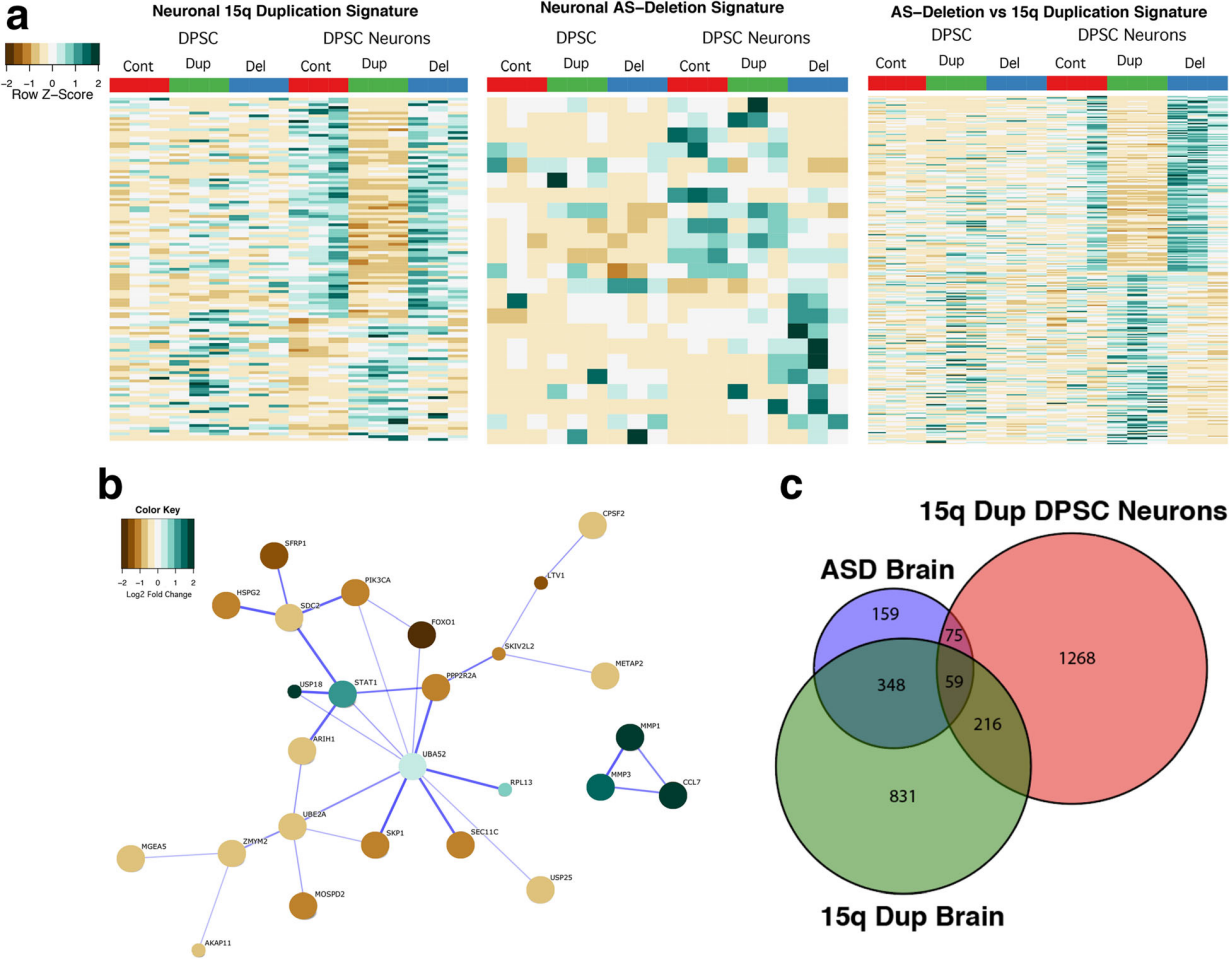
Global gene expression signatures for Dup15q and Angelman syndromes. **a** Unsupervised clustering heatmap signatures in DPSC neuronal cultures for 15q Duplication vs controls (left), AS deletion vs controls (middle), and AS deletion vs 15q Duplication (right). Note that ~ 2/3 of the transcripts went down in the DPSC neuronal cultures in 15q Duplication vs control and approximately 50% of the transcripts decreased in duplication vs AS deletion DPSC-neurons. **b** STRING analysis of significantly different transcripts in DPSC neuronal cultures from 15q Duplication vs control analysis. Colors of the circles represent the Log2 fold change in gene expression. Note the strong decrease in FOXO1 gene expression and the relationship of this protein to other proteins in the significant network. Also note that three cytokines were significantly elevated at the transcript level (CCL7, MMP1, and MMP3). **c** Venn diagram showing the significant overlap among genes upregulated in Dup15q postmortem cortex, idiopathic ASD postmortem cortex, and the 1500 most significantly upregulated genes in DPSC neurons

Unsupervised cluster analysis revealed underlying gene expression signatures of differentially regulated gene sets indicating that for the 15q duplication samples, there is significant downregulation of 2/3 of the differentially expressed genes and upregulation for about 1/3 of the genes identified compared to neurotypical controls. No obvious pattern was identified in either DPSC or DPSC neuronal cultures for AS deletion samples (Fig. 3a). When we compared 15q duplication to AS deletion samples, there was an inverse relationship for 285 genes that are differentially regulated between 15q duplication and AS deletion neurons (adj. *p* value ≤ 0.05). As expected, five of these differentially regulated genes were in the duplicated/deleted region (*CYFIP1*, *UBE3A*, *HERC2*, *NIPA1*, and *TUBGCP5*). However, another 51 genes were differentially expressed between 15q duplication and neurotypical control samples as well. These results indicate that for almost half of the genes determined to show increased/decreased expression in DPSC neuronal cultures (120), the expression signature between AS deletion and neurotypical controls was not significantly different. In addition, these changes in gene expression are not just for genes within the duplicated region, but our results show significant changes in gene expression for genes outside of the 15q11.2-q13.1 region as well in 15q duplication neuronal cultures.

We tested the null hypothesis that the gene expression trends observed were random or independent of the disease state (i.e., control vs Dup15q vs AS). In both cases, chi-squared testing revealed that the downregulation of gene expression is significant in 15q duplication neuron cultures. Of the 123 genes differentially expressed in the 15q duplication disease state vs control, 44 genes were upregulated and 79 genes were downregulated. A chi-squared fit test revealed that the number of up- and downregulated genes was *not* equally represented (*p* value = .6 × 10^−3^). These results indicate that there are more significantly downregulated than upregulated genes in the 15q duplication neuronal cultures that did not occur by chance alone.

### Network analysis of differentially regulated Dup15q genes

Since there was a larger set of genes differentially regulated between Dup15q and control neurons (120) than AS and controls (20), we focused on more detailed studies of the relationships among the proteins encoded by genes up- or downregulated in the Dup15q vs control neurons (Fig. 3a). Using STRING analysis, we identified a core network of 23 proteins predicted to interact physically or through experimental evidence with the UBA52 protein providing the central link to several other proteins mostly downregulated in Dup15q neurons vs controls including the transcription factor FOXO1 and the E2 ubiquitin ligase UBE2A (Fig. 3b). A second cluster of three proteins that were significantly upregulated *F*_c_ ≥ + 2.8 fold was also identified that included the secreted immune system factors CCL7, MMP3, and MMP1 (Fig. 3b). These cytokines may play a role in the differentiation process from DPSC to DSPC-neurons, but subsequent analysis of peptide cytokine levels in the media of mature DPSC neuronal cultures failed to recapitulate the gene expression findings (data not shown).

### Gene set enrichment analysis (GSEA) of differentially regulated genes

**Fig. 4:**
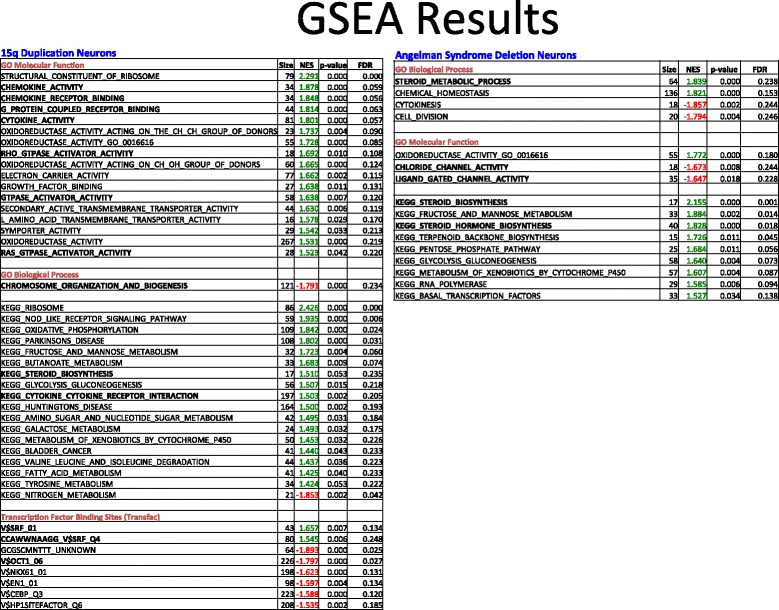
Summary of GSEA results at FDR *q*-value ≤ 0.25. The left side of table shows elevated chemokine/cytokine activity as well as both Rho and Ras G-coupled protein receptor activity. Also of note, a trending increase in transcripts responsible for steroid hormone biosynthesis and a decrease in transcripts involved in chromosomal organization. There is also a noted decrease in transcripts with OCT1 binding sites and a general increase in transcripts that respond to serum response factor (SRF). In the case of AS DPSC neuronal cultures, there was a noted increase in steroid metabolic processes and biosynthesis of steroid hormones (right table), but a general decrease in genes classified as having ligand gated and chloride channel activity. A complete list of all GSEA analysis is available in Additional file 1: Tables S1–S9

Next, we looked for Gene Ontology (GO), Kyoto Encyclopedia of Genes and Genomes (KEGG), and TRANScription FACtor Database (TRANSFAC) enhanced subsets of differentially regulated genes using a rank ordered gene lists for each binomial comparison (rank ordered by *p* value lowest to highest) for each comparison. To prioritize this data, we used GSEA software that compares two gene expression data sets for two phenotypes at a time to evaluate the presence of significant enrichments for a particular molecular signature from pre-defined Molecular Signature Database sets (MSigDB) (for details, see the “Methods” section and [38]). We looked for enrichments in KEGG, GO sub-groups, and transcription factor binding site enrichments (TRANSFAC) in DPSC neuronal cultures from both AS and Dup15q individuals as compared to typical controls (Fig. 4).

Perhaps, as expected for the AS deletion samples, since very few genes were differentially regulated, there were only a few pathway enrichment results that approached the significance level (FDR *q*-value ≤ 25%), considered a loose FDR cutoff value. Also of note, there was a significant upregulation for genes that encode steroid biosynthetic pathways (KEGG: “Steroid Biosynthesis” FDR *q*-value ≤ 0.07% and “Steroid Hormone Biosynthesis” FDR *q*-value≤ 1.8%; GO: “Steroid Metabolic Process” FDR *q*-value≤ 24%) and downregulation for genes involved in neural activity like “Chloride Channels” FDR *q*-value≤ 24% and “Ligand Gated Channels” FDR *q*-value≤ 23%.

In the Dup15q sample analysis, we identified pathways that were both significantly upregulated and downregulated by this duplication in DPSC-neurons. Consistent with the finding that the cytokine CCL7 showed a > 2 fold increase in gene expression in Dup15q neurons vs controls (adj. *p* value = 2.6 × 10^−3^), we found a significant enrichment for cytokine/chemokine activity in the Dup15q samples (FDR *q*-value ≤ 6% for GO: “Chemokine Activity,” “Chemokine Receptor Binding,” “Cytokine Activity,” and KEGG: Cytokine Cytokine Receptor Interaction FDR *q*-value ≤ 21%). There was also an increase in Ras/Rho GTPase activity genes (GO: “RAS GTPase activity” FDR *q*-value ≤ 25%, “Rho GTPase Activity” FDR *q*-value ≤ 11%, and “GTPase Activator Activity” FDR *q*-value ≤ 12%). Although not as pronounced as the 2.16 net enrichment score in “Steroid Biosynthesis” identified in AS deletion neurons, there was still a significant 1.51 net enrichment score for these transcripts in the Dup15q neurons as well.

Transcriptional downregulation between Dup15q neurons and controls proved much more interesting. First, there was a general downregulation for genes involved in chromatin organization and biogenesis (GO: FDR *q*-value ≤ 24%). Although they did not reach statistical significance, there were other downregulated genes involved in this process as well in the nominal *p* value range of ≤ 1% (notably GO: “Chromatin Assembly and Disassembly” and “Establishment or Maintenance of Chromatin Architecture” nominal *p* values ≤ 0.05). Second, there was a significant enrichment for genes potentially regulated by transcription factors OCT1 and CEBP in the downregulated set (FDR *q*-value < 2.7% and < 12% respectively). In addition, there was a nominal enrichment for genes with FOXO1 regulatory binding sites (*p* value = 7.0 × 10^−3^), a transcription factor encoding gene which was significantly downregulated in the Dup15q neurons (adj. *p* value = 3 × 10^−3^; *F*_c_ = − 3.5). The complete list of GSEA enrichment results is available in Additional file 1: Tables S1–S9.

**Fig. 5:**
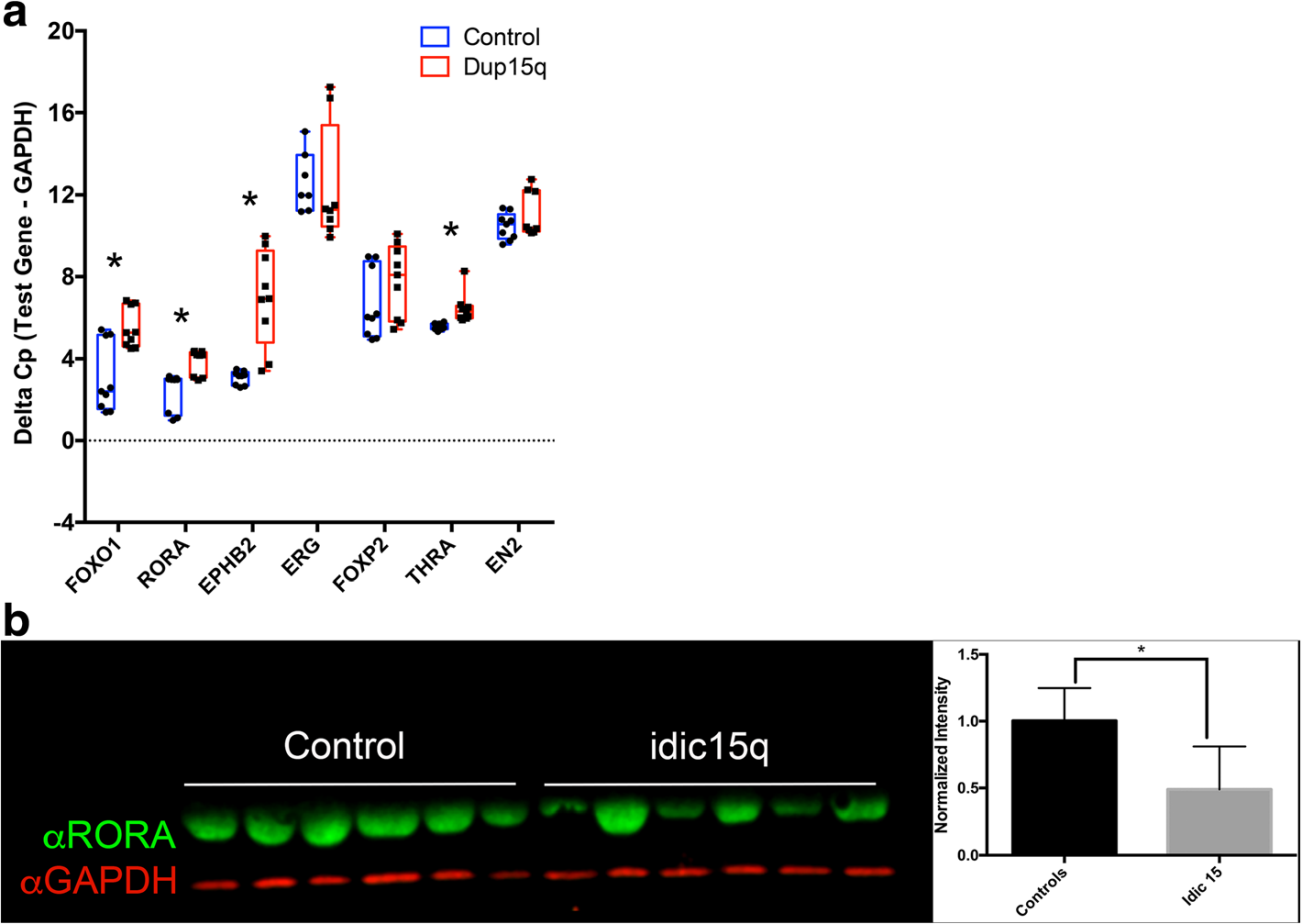
Downregulation of FOXO1 regulated transcripts EPHB2 and RORA. **a** Using a list of putative FOXO1 regulated genes from TRANSFAC, we looked for a set of genes known to be associated with ASD. While FOXP2, EN2, and ERG were not significantly downregulated by decreased FOXO1 levels in an independent cohort of idic15 DPSC neurons, we found a significant decrease in four transcripts including EPHB2 and RORA (as indicated by an increase in the average delta cross point values). Experiments were performed on six idic15 and six neurotypical controls using three technical replicates. Significance was determined using Student’s *t* test. **b** Quantitative Western blot analysis indicates a significant decrease in RORA protein levels (green) in idic15 samples vs controls. Experiments was performed three times on six samples per group. Significance was determined using an unpaired Student’s *t* test across all data points for control vs idic15 for normalized RORA levels (normalized to GAPDH in red)

We chose to validate by qRT-PCR putative FOXO1 regulated transcripts from the enrichment list which are also associated with ASD (Additional file 1: Table S7). Two genes, *RORA* and *EPHB2*, showed significantly decreased transcript expression in three unrelated idic15q DPSC neuron lines vs controls (Fig. 5a). In addition, FOXO1 showed a threefold decrease in gene expression in idic15q vs controls consistent with the RNAseq results. These results indicate a significant downregulation for FOXO1 and two autism-associated genes with FOXO1 binding sites (*RORA* and *EPHB2*) in DPSC-derived neurons (Fig. 5a). Using an antibody against the retinoid-related orphan receptor alpha (RORA) protein, we found that this receptor is significantly downregulated in neurons from an independent cohort of six idic15q subjects vs neurotypical controls (Fig. 5b). These results indicate that downregulation of the FOXO1 transcription factor may be driving a portion of the ASD phenotype in these individuals.

**Fig. 6:**
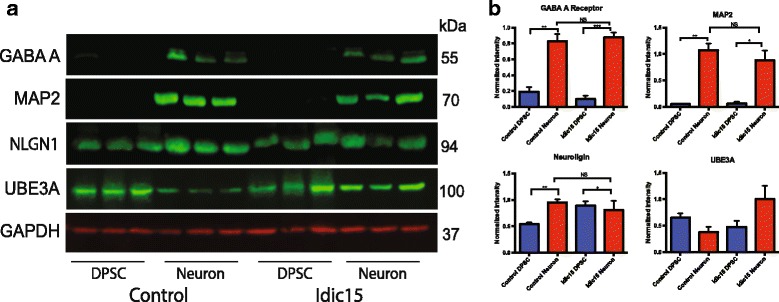
Validation of neuronal differentiation markers in idic15 neurons. Quantitative Western blots were run using total protein extracted from DPSC and DPSC-neuron cultures. **a** The western blots were probed with antibodies against the neuronal markers MAP2, GABAA, and NLGN1 as well as for UBE3A protein levels (green) and normalized to levels of GAPDH loading control (red). **b** Quantification of Western blots. For all antibodies, no significant difference was seen between control neurons and Idic15 DPSC-neurons indicating that both groups differentiate to neurons with similar efficiency. In addition, both MAP2 and GABAA were only detected in differentiated DPSC-neurons. UBE3A was detected in all lanes but appears to be expressed at higher levels in idic15 vs control DPSC-neurons, although these levels did not reach significance. Significance was determined by unpaired *t* tests between DPSC and DPSC-neurons as well as across genotypes for a given cell type (*N* = 3)

In addition, we performed quantitative Western blot analysis for known neuronal differentiation markers in both control and idic15 DPSC and DPSC-derived neurons. We found that the neuronal markers MAP2 and GABAA receptor were not expressed in DPSC but strongly expressed in DPSC-derived neurons regardless of disease state and that the marker NLGN1 was expressed in both DPSC and neurons (Fig. 6). Although we did detect the post synaptic density marker PSD95 in both control and idic15 neurons, we were unable to quantify this result due to a cross reactive band in the DPSC extracts (Additional file 2: Fig. S3). Finally, we detected UBE3A protein in both control and idic15 neurons as well as DPSC. Although the levels of UBE3A protein did not reach significance, they did appear elevated in idic15 neurons as compared to control DPSC-derived neurons (Fig. 6). These results indicate that differentiation into cells expressing neuronal differentiation markers is not inhibited in the disease state in our system.

### Gene expression in 15q duplication DPSC derived neurons correlates to gene expression in Dup15q and ASD postmortem cortex samples

As Dup15q syndrome is a common cause of ASD, which is known to have a convergent molecular pathology [46], we compared our gene expression results with postmortem gene expression data in a recently published RNA-seq study of both Dup15q and idiopathic ASD cases compared to control brain [39] (Fig. 3c). Indeed, for genes differentially expressed in the current study, we found significant enrichments in the overlaps between the genes most increased in expression in DPSC neurons from Dup15q subjects and genes increased in expression in both Dup15q syndrome and idiopathic ASD postmortem cortices (Table 3). The overlaps were stronger, and the functional enrichments more coherent, for Dup15q than for idiopathic ASD. The only terms significantly enriched (Bonferroni-corrected *p* < 0.05) in both the upregulated Dup15q postmortem + upregulated Dup15q DPSC comparison and the upregulated idiopathic ASD postmortem + upregulated Dup15q DPSC comparisons were the rather broad “response to oxygen-containing stimulus” and “response to organic substance.” In contrast, genes in the intersection of just the Dup15q comparisons had more interpretable functional enrichments such as “structural constituent of ribosome” (fold enrichment > 5; *p* = 3.45 × 10^−20^) (Additional file 1: Table S8). Taken together, these comparisons may suggest that Dup15q DPSC neurons model at least some aspects of the gene expression signature in postmortem cortex in both Dup15q syndrome and idiopathic ASD.

**Table 3:** Changes in dup15q DPSC neurons overlap changes seen in postmortem cortex in both dup15q and idiopathic ASD

Fold enrichment (*P* value)		↑ in Dupl5q cortex	↓ in Dupl5q cortex	↑ In idiopathic ASD cortex	↓ In idiopathic ASD cortex
All genes	↑ 1500 in DPSC neurons	1.41 (le-6)	1.06 (0.5)	1.25 (0.05)	1.14 (0.3)
	↓ 1500 in DPSC neurons	0.87 (0.1)	0.95 (0.6)	0.97 (0.8)	1.07 (0–6)
All genes except 15qll-13 genes	↑ 1500 in DPSC neurons	1.37 (le-5)	1.07 (0.4)	1.26 (0.04)	1.09 (0.5)
	↓ 1500 in DPSC neurons	0.88 (0.1)	0.95 (0.6)	0.96 (0.8)	1.08 (0.5)

In each cell is the fold enrichment of the overlap between the gene sets denoted by the corresponding row and column, and the two-tailed *p* value is given in parenthesis (computed using the Fisher’s exact function in scipy.stats). Columns denote genes significantly differentially expressed in postmortem cortex between either Dup15q and controls or idiopathic ASD and controls at a False Discovery Rate of 5%. Rows denote the 1500 most upregulated genes by *p* value and the 1500 most downregulated genes by *p* value in DPSC neurons comparing Dup15q DPSC neurons to control DPSC neurons (this was chosen to reduce type II error given the limited power of the DESeq analyses on these sample sizes). This is repeated excluding all genes in the 15q11-13 cytobands (which also excludes some genes flanking the duplicated region). The background was the intersection of genes expressed in Parikshak et al. and genes assigned a *p* value by DESeq in this study

## Discussion

DPSC are an excellent in vitro model to study gene expression and even protein expression changes in neurogenetic disorders. They are easily accessible through noninvasive methods and can be shipped from long distances directly to the laboratory [30]. As with any in vitro model, it is not perfect. In the near future, it will be crucial to develop protocols to differentiate these cells into specific neural subtypes coupled with extensive molecular and morphological characterization. That said, it was recently shown that gene expression profiling in neurons derived from iPSC is quite similar to neurons from DPSC and that genes involved in neural development and neurological disorders appear to be conserved in terms of regulation [3]. In addition, the epigenetic landscape of DPSC is more similar to embryonic stem cells than other stem cell types used for neurogenetics research, including iPSC [6]. DPSC-derived neurons in culture are heterogeneous with a mixed of neurons and glia cells. After maturation, there is an increase in neuronal markers, also demonstrated here in Fig. 6, and a decrease in glia markers (4,6); however, the proportion of these cells can vary from culture to culture. In this study, we took a new approach to the evaluation of gene expression in neurons from individuals with two distinct but genomically related neurodevelopmental disorders: Angelman syndrome (AS) deletion (missing one copy of the region), interstitial duplication of 15q11.2-q13.1 (a single extra copy of the region), and the isodicentric duplication of 15q (two extra copies of the region). Here, we perform the first direct comparison of gene expression in 3-week-old live neurons from subjects with duplication vs deletion of the 15q region, both of which can include autism spectrum disorder as part of the phenotype [26, 47]. Previous studies indicated that gene expression in 15q Duplication syndrome lymphoblasts shared a general expression signature with both Fragile X syndrome and idiopathic autism cases perhaps linked by the *CYFIPI* gene, a gene we also found elevated in DPSC neurons from idic(15) subjects [29]. In another study, gene expression analysis using post mortem brain tissue indicated that at least for genes within the duplicated region (most notably *GABRB3* and *UBE3A*) that expression correlates with copy number [48]. Here, we were able to confirm that copy number correlates significantly with gene expression in the PWS/AS critical region for both Angelman deletion and 15q duplication syndromes (Table 2). The upward trend between copy number and gene expression was significant for two important genes in neurons: *UBE3A*, a gene encoding the HECT domain ubiquitin protein ligase responsible for the key features of the AS phenotype [13], and a related HECT domain protein HERC2, a protein which also has been shown to physically interact with and functionally enhance the activity of UBE3A [49, 50].

One limitation in interpreting these studies is the observation that gene expression of *UBE3A* is still detected in 3-week-old DPSC neuronal cultures that came from maternal AS deletion subjects. There may be several explanations for this phenomena: (1) we were not able to use for gene expression studies DPSC neuronal cultures matured past ~ 5 weeks for gene expression studies because as these cultures mature, the number live neurons decreases; (2) DPSC are neural crest-derived cells from a lineage that may not show imprinted gene expression for UBE3A under the current culturing conditions, although this will have a stronger effect on interpretation of the AS deletion results than the Dup15q results since UBE3A duplications are primarily maternal in origin and therefore *UBE3A* will be expressed on the duplicated chromosome; (3) the proportion of glial cells, which biallelically express *UBE3A* in humans, to neurons is high in cultured DPSC neurons, masking the mono-allelic expression of *UBE3A* in neurons [51]. All of these possibilities will require further investigations using more mature DPSC neuron cultures, differing culture conditions, and possibly a shift to the use of induced dental pulp stem cells, if needed, as in a recent ASD study [52].

The coordinated gene expression for *UBE3A* and *HERC2*, located within the duplicated/deleted region on 15q, may have a significant impact on the phenotypes in both AS and 15q duplication syndromes. In the case of AS, the majority of individuals with this syndrome have a deletion (70%), and among the deletion cases, the BP2-BP3 deletion is more frequent (50%) but still includes both *UBE3A* and *HERC2* [14]. The other ~ 15% of individuals have imprinting center defects, mutations in maternal *UBE3A*, or paternal uniparental disomy. Individuals with a *UBE3A* mutations have a milder AS phenotype with severe developmental delay, absence of speech, inappropriate laughter and EEG abnormalities as the most consistent findings and ataxia, epilepsy and microcephaly can present as mild or even absent [53, 54], suggesting that loss of *UBE3A* expression in neurons alone is not enough to recapitulate all aspects of the AS phenotype. In fact, regulation of UBE3A by HERC2 in deletion cases may enhance the phenotype substantially and could certainly play a role in affecting UBE3A function not only in non-imprinted tissues but also in tissues where a preference for maternal-specific expression has been found like heart and liver [55] or even in parts of the brain where there is biallelic expression for *UBE3A* in GFAP positive cells lining the lateral ventricles where neural stem cells develop [56]. In fact, mutations in HERC2 can correlate with an Angelman-like phenotype clearly implicating the physical interaction between HERC2 and UBE3A in some aspect of the syndrome [57]. Given that HERC2 mutations can also cause phenotypes on the autism spectrum in some cohorts [58], we propose that the elevated levels of gene expression for both UBE3A and HERC2 found here in neurons from 15q Duplication syndrome subjects may have a more profound impact on the autism phenotype in this disorder than previously considered [7].

With regard to the 15q Duplication syndrome, this study provides important clues to the underlying molecular etiology of the disorder in patient-derived neurons. Gross downregulation of developmental transcription factors such as *FOXO1* (*F*_c_ = − 3.4), *HAND2* (*F*_c_ = − 7.3), and *SOX11* (*F*_c_ = − 4.7) in 15q duplication neurons may have significant pleiotropic effects on other genes regulated by these transcription factors. We identified an enrichment for the downregulation of genes with FOXO1 binding sites (nominal *p* value = 7X10^− 3^), for example, that implicates genes regulated by FOXO1 in the syndrome. These genes include proteins involved in chromatin reorganization as well as some specific genes involved in regulating action potentials and dendrite formation. Indeed, there is specific downregulation of both EPHB2 and RORA, two potential FOXO1 gene targets, at the transcript level (Fig. 6a). The validation of downregulation of RORA protein in an independent set of idic15q DPSC neuron cultures vs controls lends further support to the idea that these transcription factors, especially FOXO1, may be the branch point for global downregulation of autism-associated nuclear receptor encoding genes like RORA in Dup15q syndrome.

Although the identification of a transcription factor like FOXO1 that may regulate several downstream ASD-related transcripts is an important finding in this study, perhaps more important was the finding that RORA protein levels are significantly decreased in idic15q vs control neurons. RORA transcript levels were previously found to be significantly decreased in blood from ASD vs control individuals [38]. Moreover, RORA, which is a nuclear hormone receptor, has been shown in neuronal cell lines to transcriptionally regulate ASD-associated genes [59]. RORA is therefore an excellent candidate for future molecular studies of autism in Dup15q syndrome, a major portion of the phenotype [26].

Moreover, these transcription factors have now been found to be dysregulated in other ASD samples. This prompted us to investigate the correlation between our gene expression data in 15q duplication neurons and a recent study looking at gene expression changes in postmortem brain samples from both Dup15q syndrome and idiopathic ASD cases. We found a clear overlap in 1/3 of upregulated genes in all three comparisons (Table 3). Contrasting DPSC-derived neurons to postmortem cortical samples should be taken with caution as our in vitro model does not consist exclusively of cortical neurons. However, these comparisons are instructive for two reasons: firstly, it shows that DPSC-derived neurons in Dup15q syndrome recapitulate the molecular changes seen in postmortem Dup15q and idiopathic autism brain, even and especially outside the 15q region and that this may therefore be a model with a tractable molecular handle to learn about neurobiology in idiopathic ASD more generally; secondly, it demonstrates that at least some components of the convergent molecular signature in autism brains are inherent to the local neurobiology and are not downstream neurological responses to growing up autistic in a neurotypical world.

It is perhaps no surprise that gene expression in AS neurons did not differ significantly from control neurons for many genes since the primary gene expected to contribute to pathogenesis in AS encodes an E3 ubiquitin ligase that targets other proteins for degradation or other cellular functions at the protein level. Several potential protein targets contributing to pathogenesis have been identified over the years, but no targets have yet emerged that can clearly explain all of the neurological phenotypes in AS on their own [16, 19, 44, 50, 60, 61]. It has been known for some time, however, that UBE3A can act as a transcriptional co-activator of steroid hormone receptors [22], and our own work in Drosophila indicates that Dube3a expression can regulate gene expression for GTP cyclohydrolase I leading to increased or decreased dopamine in the brains of flies [15]. Here, we show that patient-derived DPSC neuronal cultures do show aberrant gene expression for 23 genes compared to controls. Of these, the one gene that does seem consistent with other UBE3A studies is the *CRY2* gene (adj. *p* value = 2 × 10^−2^; F_c_ = − 2.0). *CRY2* is a circadian clock gene, and circadian rhythm defects have been found in both fly and mouse models of AS [44, 45]. It remains to be seen if this regulation of *CRY2* is contributing to these circadian defects, however, and the limited number of transcriptional changes found in the AS neurons seems to imply that transcriptional regulation of genes other than *UBE3A* is not a key contributor to the pathogenesis of AS in neurons.

## Conclusions

In conclusion, this study demonstrates that DPSC-derived neurons express several neuronal differentiation markers (MAP2, GABAA, NLGN1, and PSD95) and may prove a useful tool for the study of neurogenetic disease. Here, we demonstrated that global gene expression in immature neurons is not significantly altered in AS deletion DPSC neurons but that changes in gene expression found in the 15q duplication DPSC neurons indicate that this particular duplication results in downregulation, rather than up, for genes located outside of the Dup15q critical region through transcription factors like HAND2, OCT1, and FOXO1 and subsequently the ASD associated transcriptional activator RORA. Finally, we found more changes in gene expression in Dup15q than AS cell lines, implying that the mechanism of AS may be primarily through the degradation of UBE3A protein substrates as previously proposed in other studies.

## Additional files

Additional file 1: Tables S1–S9. Complete GSEA analysis for all samples and FOXO1 and functional enrichment analysis of ASD brain vs Dup15q DPSC neurons. S9 is a table of primers used for qRT-PCR. (XLSX 68 kb)

Additional file 2: Figure S1. Copy number and breakpoint analysis by FISH. Examples of each cell line are shown. All cell lines were analyzed with NIPA1 + SNRPN or TRPM1 + SNRPN. At least 15 cell preparations were analyzed for each line to determine the breakpoints and copy numbers. Note, DPSC line TP-041 is an interstitial duplication line with three copies of the BP2-BP3 region (green signals) but only one copy of the BP1-BP3 or BP4-BP5 regions (red signals). Fig. S2 Boxplots for specific genes of interest mentioned in the text. These are the RPKM averages across all three biological replicates for DPSC and DPSC neuronal cultures and for control (red), Dup15q (blue), and AS (green) for each gene of interest. Error bars are standard deviation. Fig. S3 PSD95 staining of DPSC and DPSC-derived neurons. A band correlating to PSD95 is seen in the DPSC-neuronal cultures. Unfortunately, a cross-reactive band also present in the α-goat secondary alone blot interfered with the signal for PSD95 in DPSC alone making quantification impossible (top versus middle lane). GAPDH was used as a loading control. (PDF 3128 kb)
