# Supplementary material for: Significant transcriptional changes in 15q duplication but not Angelman syndrome deletion stem cell-derived neurons
Source: Mol Autism. 2018 Jan 27;9:6. doi: 10.1186/s13229-018-0191-y (PMC5787244; doi:10.1186/s13229-018-0191-y)
Supplement: Supplementary file 2 — Copy number and breakpoint analysis by FISH. Examples of each cell line are shown. All cell lines were analyzed with NIPA1 + SNRPN or TRPM1 + SNRPN. At least 15 cell preparations were analyzed for each line to determine the breakpoints and copy numbers. Note, DPSC line TP-041 is an interstitial duplication line with three copies of the BP2-BP3 region (green signals) but only one copy of the BP1-BP3 or BP4-BP5 regions (red signals). Fig. S2 Boxplots for specific genes of interest mentioned in the text. These are the RPKM averages across all three biological replicates for DPSC and DPSC neuronal cultures and for control (red), Dup15q (blue), and AS (green) for each gene of interest. Error bars are standard deviation. Fig. S3 PSD95 staining of DPSC and DPSC-derived neurons. A band correlating to PSD95 is seen in the DPSC-neuronal cultures. Unfortunately, a cross-reactive band also present in the α-goat secondary alone blot interfered with the signal for PSD95 in DPSC alone making quantification impossible (top versus middle lane). GAPDH was used as a loading control. (PDF 3128 kb) [file 13229_2018_191_MOESM2_ESM.pdf]

Figure S1

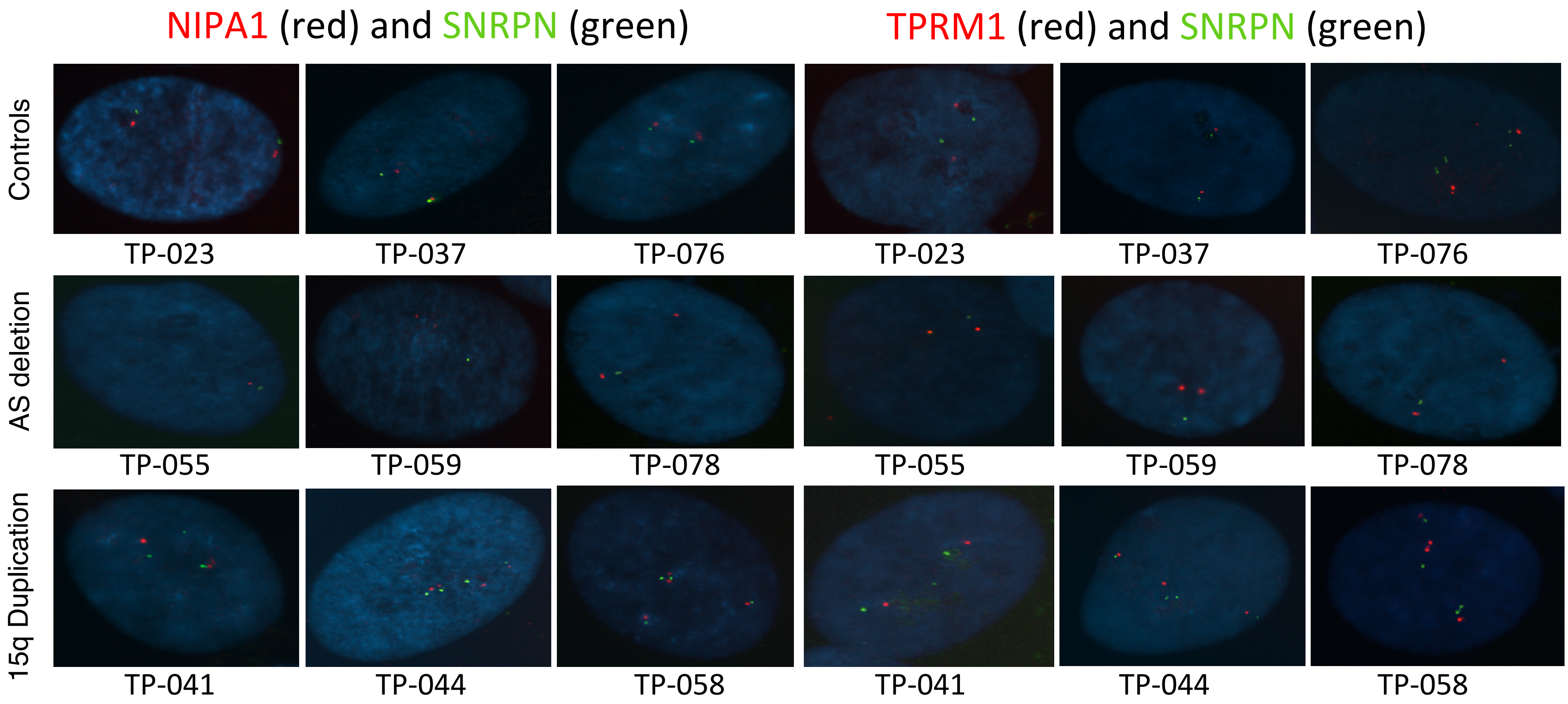
NIPA1 (red) and SNRPN (green)
TPRM1 (red) and SNRPN (green)
Controls
AS deletion
15q Duplication
TP-023
TP-037
TP-076
TP-023
TP-037
TP-076
TP-055
TP-059
TP-078
TP-055
TP-059
TP-078
TP-041
TP-044
TP-058
TP-041
TP-044
TP-058

Figure S2

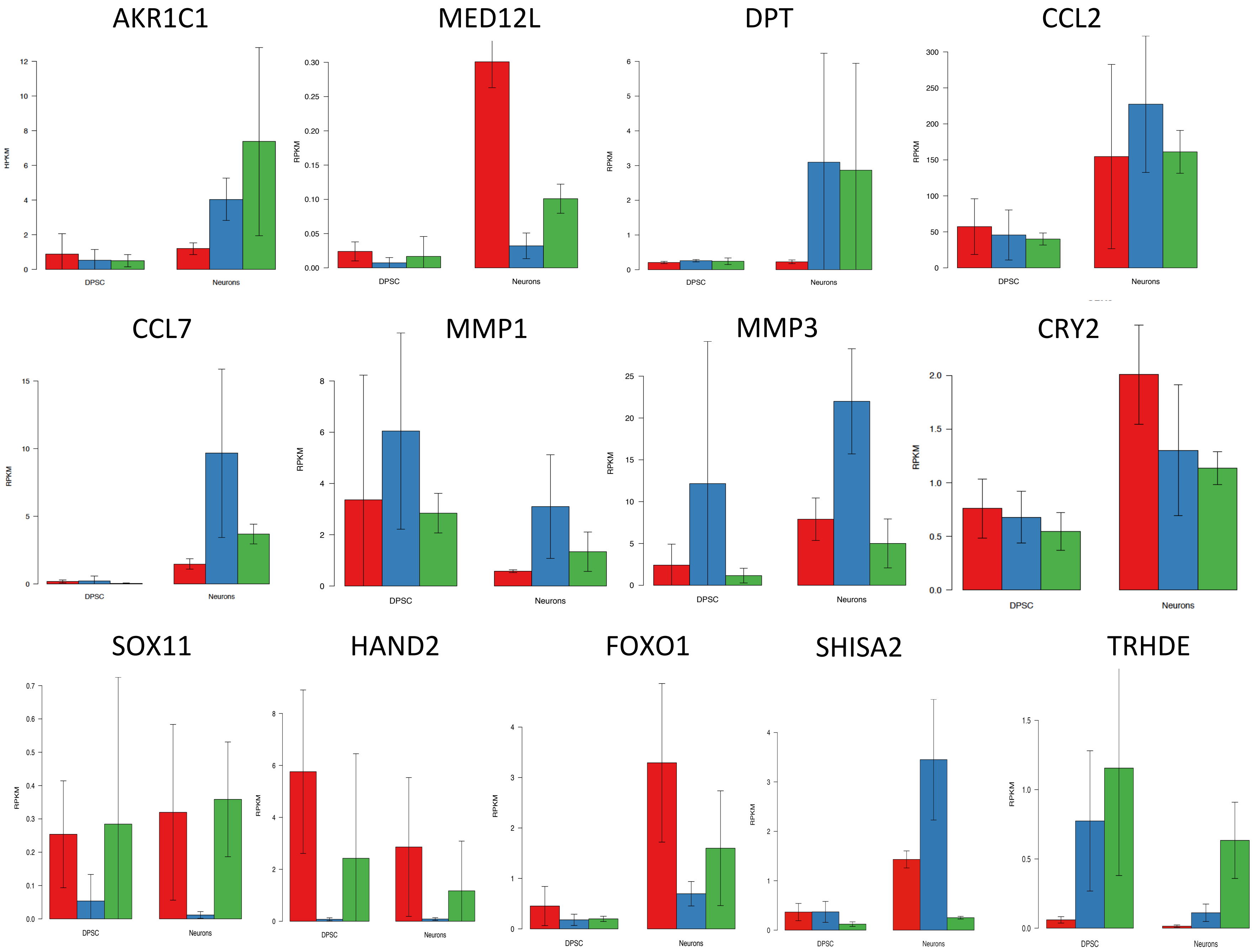
AKR1C1
MED12L
DPT
CCL2
CCL7
MMP1
MMP3
CRY2
SOX11
HAND2
FOXO1
SHISA2
TRHDE
RPKM
DPSC
Neurons

Figure S3

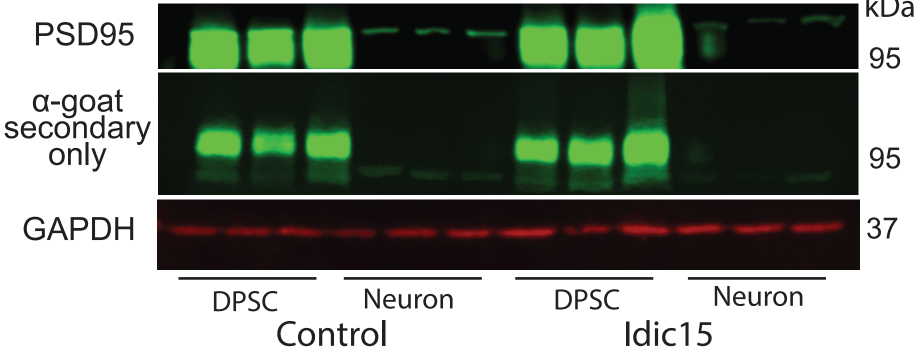

PSD95
α-goat secondary only
GAPDH
kDa
95
95
37
DPSC
Neuron
DPSC
Neuron
Control
Idic15

TRANSFAC: 15q Duplication Neurons

DOWN

NAME	SIZE	ES	NES	NOM p-val	FDR q-val	FWER p-val
GCGSCMNTTT_UNKNOWN	64	-0.5094314	-1.8933098	0	0.02501921	0.017
V$OCT1_06	226	-0.3933881	-1.7968041	0	0.02733338	0.035
V$NKX61_01	198	-0.3649626	-1.6230397	0	0.13079427	0.23
V$EN1_01	98	-0.4000299	-1.5965902	0.00420168	0.13397652	0.301
V$CEBP_Q3	223	-0.3518449	-1.5878625	0	0.11962409	0.331
V$HP1SITEFACTOR_Q6	208	-0.3452416	-1.5349915	0.0021645	0.18466899	0.516
V$MYOGNF1_01	39	-0.4300448	-1.4831299	0.02626263	0.27976456	0.743
V$GATA1_01	221	-0.322842	-1.4529176	0	0.33564568	0.85
V$SOX9_B1	217	-0.3191878	-1.4236258	0.0046729	0.41007605	0.934
V$SREBP_Q3	218	-0.3090884	-1.4095014	0.00888889	0.43028527	0.96
V$OCT1_07	141	-0.3329824	-1.4077808	0.01545254	0.39733857	0.96
YGCANTGCR_UNKNOWN	119	-0.3381355	-1.4044033	0.02118644	0.380837	0.968
V$DR3_Q4	124	-0.3318088	-1.3932549	0.01476793	0.39827082	0.981
MYAATNNNNNNNGGC_UNKNOWN	99	-0.3469147	-1.3911699	0.02296451	0.3774542	0.983
V$VDR_Q3	192	-0.3127211	-1.3895535	0.00445434	0.35847512	0.985
V$SRY_02	229	-0.3060061	-1.3804193	0.01106195	0.37415162	0.992
V$AP3_Q6	217	-0.3025683	-1.3705543	0.00631579	0.3910617	0.993
V$E2F_Q3	214	-0.3045821	-1.3676448	0.00909091	0.3813685	0.994
V$EVI1_03	46	-0.3891891	-1.3647718	0.0533049	0.37119076	0.995
V$SMAD3_Q6	203	-0.3031859	-1.3590547	0.01310044	0.3741324	0.997
V$AML1_Q6	240	-0.2956955	-1.3540615	0.01809955	0.3729231	0.998
V$NKX22_01	158	-0.317044	-1.3533521	0.02672606	0.3583222	0.998
V$HFH1_01	215	-0.3021202	-1.3487269	0.0087146	0.36005485	0.998
V$AML1_01	240	-0.2956955	-1.3477187	0.0137931	0.3483203	0.998
V$FOXO1_02	215	-0.2949153	-1.3463267	0.00697674	0.33836308	0.998
V$RP58_01	178	-0.3019264	-1.3356836	0.02252252	0.36630166	0.999
V$CHX10_01	191	-0.298927	-1.333554	0.01492537	0.3599701	0.999
RRAGTTGT_UNKNOWN	230	-0.2960938	-1.3325471	0.01327434	0.3508565	0.999
V$HIF1_Q3	208	-0.2936804	-1.3314378	0.015625	0.34252292	0.999
TGACGTCA_V$ATF3_Q6	211	-0.2929922	-1.3294653	0.0046729	0.33794484	0.999
V$MEIS1AHOXA9_01	97	-0.3268906	-1.3261049	0.02553191	0.3381378	1

UP

NAME	SIZE	ES	NES	NOM p-val	FDR q-val	FWER p-val
V$SRF_01	43	0.48542336	1.6573973	0.00718133	0.13352197	0.187
CCAWWNAAGG_V$SRF_Q4	80	0.40319514	1.5448364	0.00567108	0.2478092	0.539
V$TATA_01	193	0.30879465	1.3548863	0.01489758	1	0.999
V$ISRE_01	225	0.30589628	1.3501171	0.01092896	1	0.999
V$NFKAPPAB_01	217	0.30308276	1.3335816	0.02169982	1	0.999
V$HOX13_01	40	0.39323503	1.32752	0.10242086	0.93175566	0.999
RGTTAMWNATT_V$HNF1_01	53	0.37206316	1.3223978	0.06990291	0.8449261	0.999
V$SRF_C	190	0.30571803	1.321815	0.02678572	0.74310005	0.999

(Highlighted) Mentioned in the Text

TRANSFAC: AS Deletion Neurons

DOWN

NAME	SIZE	ES	NES	NOM p-val	FDR q-val	FWER p-val
V$OCT1_07	144	-0.3807221	-1.5705194	0	0.7485122	0.407
V$EVI1_03	49	-0.4583496	-1.5698875	0.01724138	0.37763244	0.41
V$IRF_Q6	227	-0.3351715	-1.4980567	0	0.5593537	0.711
V$ICSBP_Q6	231	-0.3313863	-1.4486657	0.002079	0.7329453	0.894
AGCYRWTTC_UNKNOWN	113	-0.3535078	-1.4262518	0.01054852	0.747513	0.944
V$MEIS1AHOXA9_01	100	-0.3584329	-1.4151071	0.02165354	0.7043254	0.965
V$EVI1_06	18	-0.4935676	-1.3844324	0.10229645	0.84814113	0.99
V$AML1_01	245	-0.3101316	-1.3788893	0.00410678	0.7864868	0.993
V$HP1SITEFACTOR_Q6	211	-0.312507	-1.3728573	0.00976563	0.7419695	0.994
V$AML1_Q6	245	-0.3101316	-1.3712174	0.00824742	0.6798666	0.994
V$AMEF2_Q6	234	-0.3065637	-1.3519431	0.01207244	0.75519454	0.997
V$HOX13_01	39	-0.4132153	-1.3484844	0.06614786	0.71743125	0.998
V$CDPCR3HD_01	215	-0.3068185	-1.3481336	0.01810865	0.6646419	0.998
V$POU3F2_01	86	-0.3556489	-1.3480694	0.04642166	0.6178575	0.998
CATTGTYY_V$SOX9_B1	338	-0.2934313	-1.3464539	0.00390625	0.58556753	0.998
V$E4BP4_01	208	-0.309348	-1.3416383	0.01782178	0.5763137	0.998
V$HFH4_01	179	-0.315409	-1.3405806	0.02647658	0.5487232	0.999
V$NKX61_01	202	-0.3068869	-1.3403333	0.01716738	0.5198254	0.999

UP

NAME	SIZE	ES	NES	NOM p-val	FDR q-val	FWER p-val
V$PAX3_01	22	0.51021683	1.497745	0.04393305	0.8342927	0.708
V$ALPHACP1_01	236	0.31215733	1.3771724	0.01639344	1	0.994
CCAATNNSNNNGCG_UNKI	54	0.39008346	1.3762366	0.05761317	1	0.994

Mentioned in the Text

GO: 15q Duplication Neurons

DOWN

NAME	SIZE	ES	NES	NOM p-val	FDR q-val	FWER p-val
CHROMOSOME_ORGANIZATION_AND_BIOGENESIS	121	-0.4339858	-1.8201128	0	0.5585529	0.482
CHROMATIN_ASSEMBLY_OR_DISASSEMBLY	26	-0.5826206	-1.7727206	0.00639659	0.47712943	0.678
ESTABLISHMENT_AND_OR_MAINTENANCE_OF_CHROMATIN_ARCHITECTURE	75	-0.4294769	-1.6466404	0.00635593	1	0.98
MACROMOLECULAR_COMPLEX_DISASSEMBLY	15	-0.6100932	-1.6214776	0.02114165	1	0.997
TRANSMEMBRANE_RECEPTOR_PROTEIN_PHOSPHATASE_ACTIVITY	18	-0.580447	-1.6134549	0.0210084	0.8977136	0.997
REGULATION_OF_ACTION_POTENTIAL	16	-0.5768601	-1.5918566	0.02459016	0.8929916	0.999
CHROMATIN_ASSEMBLY	16	-0.5808365	-1.5697571	0.03131524	0.90862095	1
GENERAL_RNA_POLYMERASE_II_TRANSCRIPTION_FACTOR_ACTIVITY	32	-0.4683348	-1.541086	0.0190678	0.9858792	1
PORE_COMPLEX	36	-0.4601928	-1.5379628	0.02760085	0.89746743	1
DNA_PACKAGING	32	-0.4644173	-1.5290788	0.01898734	0.8651769	1
DENDRITE	15	-0.5660704	-1.51138	0.04462475	0.8930051	1
PEROXISOME_ORGANIZATION_AND_BIOGENESIS	16	-0.5441632	-1.4997828	0.06029106	0.89454967	1
PROTEIN_AMINO_ACID_DEPHOSPHORYLATION	59	-0.3967321	-1.4777185	0.02985075	0.97494745	1
SEQUENCE_SPECIFIC_DNA_BINDING	55	-0.4070704	-1.4746774	0.01918977	0.9252741	1
NUCLEAR_MEMBRANE	48	-0.4211148	-1.4667499	0.03416856	0.91236836	1
NUCLEAR_PORE	31	-0.440474	-1.4466326	0.04365904	0.9843774	1

UP

NAME	SIZE	ES	NES	NOM p-val	FDR q-val	FWER p-val
STRUCTURAL_CONSTITUENT_OF_RIBOSOME	79	0.60831857	2.3000758	0	0	0
CHEMOKINE_ACTIVITY	34	0.5833463	1.884044	0	0.13948384	0.228
CHEMOKINE_RECEPTOR_BINDING	34	0.5833463	1.8780724	0	0.10352428	0.246
LIPID_HOMEOSTASIS	15	0.6839274	1.8327537	0.00385356	0.1398569	0.406
CYTOKINE_ACTIVITY	81	0.47130308	1.8297948	0	0.11460169	0.409
G_PROTEIN_COUPLED_RECEPTOR_BINDING	44	0.5296605	1.8295625	0	0.09586461	0.409
RESPONSE_TO_OXIDATIVE_STRESS	43	0.5205627	1.7810241	0.00579151	0.15431596	0.611
CHEMICAL_HOMEOSTASIS	134	0.42244628	1.7672718	0	0.15933107	0.659
OXIDOREDUCTASE_ACTIVITY_ACTING_ON_THE_CH_CH_GROUP_OF_DONORS	23	0.5848808	1.7657856	0.00773694	0.14387622	0.665
OXIDOREDUCTASE_ACTIVITY_GO_0016616	55	0.48507896	1.7547	0	0.14627519	0.711
RHO_GTPASE_ACTIVATOR_ACTIVITY	18	0.6055668	1.6716973	0.00968992	0.31963742	0.954
ELECTRON_CARRIER_ACTIVITY	77	0.43772656	1.6659968	0.0019084	0.30756313	0.963
REGULATION_OF_CYCLIN_DEPENDENT_PROTEIN_KINASE_ACTIVITY	41	0.49528235	1.6654005	0.00180832	0.28580397	0.964
DEVELOPMENT_OF_PRIMARY_SEXUAL_CHARACTERISTICS	21	0.57378125	1.6525108	0.01142857	0.30238238	0.98
GROWTH_FACTOR_BINDING	27	0.5301724	1.6514517	0.00598802	0.2844534	0.98
OXIDOREDUCTASE_ACTIVITY_ACTING_ON_CH_OH_GROUP_OF_DONORS	60	0.4556028	1.6512581	0	0.26723757	0.98
VESICLE_MEMBRANE	28	0.52973676	1.6331445	0.00369004	0.29772532	0.994
SECONDARY_ACTIVE_TRANSMEMBRANE_TRANSPORTER_ACTIVITY	44	0.4733048	1.6209818	0.00584795	0.3127635	0.995
RESPONSE_TO_EXTERNAL_STIMULUS	263	0.3569134	1.6198318	0	0.29943386	0.995
WOUND_HEALING	46	0.46991926	1.6192056	0.00948767	0.28596547	0.995
GTPASE_ACTIVATOR_ACTIVITY	58	0.44672424	1.6129533	0.00377359	0.2888022	0.997

(yellow)	Mentioned in the Text
(green)	Also of interest

GO: AS Deletion Neurons

DOWN

NAME	SIZE	ES	NES	NOM p-val	FDR q-val	FWER p-val
CYTOKINESIS	18	-0.6795768	-1.8728243	0.00206186	0.3198453	0.251
CELL_DIVISION	20	-0.6347529	-1.8209481	0	0.30862626	0.443
REGULATION_OF_PEPTIDYL_TYROSINE_PHOSPHORYLATION	16	-0.6616356	-1.7688583	0.00194932	0.38295674	0.646
CELL_CELL_ADHESION	79	-0.4531722	-1.7102556	0	0.5399729	0.859
CELLULAR_MORPHOGENESIS_DURING_DIFFERENTIATION	46	-0.4996825	-1.6966162	0.01022495	0.48838636	0.896
GROWTH_FACTOR_ACTIVITY	47	-0.4905852	-1.6842574	0.00202429	0.46317023	0.926
ANION_CHANNEL_ACTIVITY	18	-0.5992384	-1.6670983	0.00814664	0.4731243	0.96
LIGAND_GATED_CHANNEL_ACTIVITY	35	-0.5119488	-1.6586397	0.01077586	0.44763425	0.965
POSITIVE_REGULATION_OF_PROTEIN_AMINO_ACID_PHOSPHORYLATION	18	-0.5910826	-1.6436394	0.01052632	0.46002585	0.983
CHLORIDE_CHANNEL_ACTIVITY	18	-0.5992384	-1.6318763	0.01778656	0.45864907	0.99
CELLULAR_RESPIRATION	19	-0.5811528	-1.6277045	0.02620968	0.43265176	0.991
CHROMATIN_ASSEMBLY_OR_DISASSEMBLY	26	-0.545148	-1.6260283	0.01361868	0.4017679	0.991
REGULATION_OF_PROTEIN_AMINO_ACID_PHOSPHORYLATION	28	-0.5269707	-1.6205647	0.00828157	0.39110622	0.992
NEURON_DEVELOPMENT	58	-0.4515381	-1.6056588	0.00788955	0.41111386	0.997
METALLOPEPTIDASE_ACTIVITY	46	-0.4605937	-1.5976261	0.00801603	0.41039008	0.998
POSITIVE_REGULATION_OF_PROTEIN_MODIFICATION_PROCESS	27	-0.5169987	-1.5852486	0.0244898	0.42801636	1
NEURITE_DEVELOPMENT	50	-0.4561331	-1.5704453	0.01039501	0.45334646	1
POSITIVE_REGULATION_OF_CELL_CYCLE	16	-0.5883299	-1.5534823	0.03017241	0.49400184	1
PEPTIDYL_TYROSINE_PHOSPHORYLATION	25	-0.5257874	-1.5464498	0.02345416	0.49399638	1
THIOLESTER_HYDROLASE_ACTIVITY	15	-0.5826979	-1.5402362	0.03250478	0.49413928	1
COLLAGEN	22	-0.5351812	-1.5391032	0.03386454	0.47478908	1
SULFURIC_ESTER_HYDROLASE_ACTIVITY	16	-0.5687776	-1.5352945	0.04	0.46625507	1
GLYCOLIPID_METABOLIC_PROCESS	16	-0.5713344	-1.5301486	0.03784861	0.46270463	1
CHROMOSOMEPERICENTRIC_REGION	31	-0.4808978	-1.5228419	0.03483607	0.47023144	1
REGULATION_OF_PROTEIN_MODIFICATION_PROCESS	42	-0.445787	-1.5141118	0.02574257	0.48172045	1
AXONOGENESIS	41	-0.4501657	-1.5005414	0.02910603	0.5139358	1

UP

NAME	SIZE	ES	NES	NOM p-val	FDR q-val	FWER p-val
STEROID_METABOLIC_PROCESS	64	0.50926155	1.8503424	0	0.39164612	0.35
CHEMICAL_HOMEOSTASIS	136	0.44306058	1.8335853	0	0.23609515	0.4
OXIDOREDUCTASE_ACTIVITY_GO_0016616	55	0.49824286	1.7802109	0	0.31021947	0.619
OXIDOREDUCTASE_ACTIVITY_ACTING_ON_THE_CH_CH_GROUP_OF_DON(	23	0.6051666	1.7617718	0.0019685	0.28931224	0.699
OXIDOREDUCTASE_ACTIVITY_ACTING_ON_CH_OH_GROUP_OF_DONORS	60	0.47402784	1.7153921	0	0.37308097	0.854
STEROID_BIOSYNTHETIC_PROCESS	22	0.57602483	1.66491	0.00804829	0.509658	0.957
NEGATIVE_REGULATION_OF_TRANSPORT	17	0.6166911	1.6592981	0.00744879	0.46184468	0.965
POTASSIUM_ION_TRANSPORT	49	0.47788802	1.6390767	0.0040568	0.4850235	0.98
VOLTAGE_GATED_POTASSIUM_CHANNEL_COMPLEX	37	0.49696887	1.6127906	0.02053388	0.5496902	0.996
OXIDOREDUCTASE_ACTIVITY_GO_0016705	31	0.51510423	1.6080852	0.01635992	0.51577073	0.997
REGULATION_OF_SECRETION	33	0.5096709	1.6061571	0.00590551	0.47715434	0.997
ATPASE_ACTIVITY_COUPLED_TO_TRANSMEMBRANE_MOVEMENT_OF_IOI	18	0.5774405	1.5980569	0.01953125	0.47134835	0.997
ION_HOMEOSTASIS	112	0.39221933	1.5854561	0.00398406	0.48894048	0.999
PROTEIN_PROCESSING	48	0.45572343	1.5844144	0.01214575	0.45853817	0.999
NEUROPEPTIDE_RECEPTOR_ACTIVITY	15	0.5987516	1.582735	0.03143419	0.4331638	0.999
CELLULAR_CATION_HOMEOSTASIS	93	0.40122604	1.5781736	0.0038835	0.42337033	0.999
OXYGEN_BINDING	19	0.5668802	1.5750759	0.02941177	0.40749937	0.999
NEUROPEPTIDE_BINDING	15	0.5987516	1.5654978	0.00976563	0.4164075	1
CARBOHYDRATE_TRANSPORT	16	0.58402526	1.5534754	0.03292181	0.4337085	1
RESPONSE_TO_NUTRIENT_LEVELS	28	0.5217431	1.5502299	0.02514507	0.4233382	1
SIGNAL_SEQUENCE_BINDING	15	0.5940841	1.549926	0.04743083	0.4040046	1
CARBOHYDRATE_TRANSMEMBRANE_TRANSPORTER_ACTIVITY	15	0.5963993	1.5387459	0.03653846	0.42123857	1
VOLTAGE_GATED_POTASSIUM_CHANNEL_ACTIVITY	33	0.4852749	1.535342	0.01945525	0.4140759	1

Mentioned in the Text

Also of interest

KEGG: 15q Duplication Neurons

DOWN

NAME	SIZE	ES	NES	NOM p-val	FDR q-val	FWER p-val
KEGG_NITROGEN_METABOLISM	21	-0.6195197	-1.8528584	0.0021692	0.04190687	0.05
KEGG_DORSO_VENTRAL_AXIS_FORMATION	23	-0.5353528	-1.5962297	0.02040816	0.31829458	0.535
KEGG_COLORECTAL_CANCER	61	-0.4084651	-1.5107552	0.01750547	0.4364343	0.796
KEGG_GLYCEROPHOSPHOLIPID_METABOLISM	68	-0.3824661	-1.4601109	0.0203252	0.48245612	0.911
KEGG_O_GLYCAN_BIOSYNTHESIS	26	-0.4429121	-1.376868	0.0755102	0.70290583	0.995
KEGG_ALANINE_ASPARTATE_AND_GLUTAMATE_METABOLISM	30	-0.4142374	-1.3466687	0.09052631	0.72519404	0.998
KEGG_PROSTATE_CANCER	86	-0.3274035	-1.3183947	0.0516129	0.74953264	1
KEGG_GLYCOSYLPHOSPHATIDYLINOSITOL_GPI_ANCHOR_BIOSYNTHESIS	24	-0.4263476	-1.2987518	0.12847966	0.73861414	1
KEGG_RENAL_CELL_CARCINOMA	68	-0.3336962	-1.2761695	0.08478261	0.75236195	1
KEGG_RNA_DEGRADATION	55	-0.3399249	-1.2471025	0.10775862	0.8043293	1
KEGG_MTOR_SIGNALING_PATHWAY	51	-0.3382346	-1.2241685	0.16059957	0.8366159	1
KEGG_PROGESTERONE_MEDIATED_OOCYTE_MATURATION	82	-0.307321	-1.2127756	0.15010571	0.8168095	1
KEGG_LYSINE_DEGRADATION	43	-0.3535678	-1.211876	0.1655914	0.757375	1
KEGG_CALCIUM_SIGNALING_PATHWAY	158	-0.2761783	-1.2018442	0.09368192	0.7449851	1
KEGG_AMINOACYL_TRNA_BIOSYNTHESIS	40	-0.3439494	-1.1756773	0.22020201	0.8042487	1
KEGG_AMYOTROPHIC_LATERAL_SCLEROSIS_ALS	46	-0.3187745	-1.1364563	0.22520661	0.9308508	1
KEGG_OOCYTE_MEIOSIS	104	-0.2766291	-1.1350114	0.18979591	0.88252515	1
KEGG_PEROXISOME	75	-0.2940089	-1.1291635	0.25211865	0.85886955	1
KEGG_CELL_ADHESION_MOLECULES_CAMS	108	-0.2709754	-1.1256946	0.21365638	0.82850426	1
KEGG_NATURAL_KILLER_CELL_MEDIATED_CYTOTOXICITY	96	-0.2784223	-1.124122	0.21318682	0.79267675	1

UP

NAME	SIZE	ES	NES	NOM p-val	FDR q-val	FWER p-val
KEGG_RIBOSOME	86	0.6251042	2.4255242	0	0	0
KEGG_NOD_LIKE_RECEPTOR_SIGNALING_PATHWAY	59	0.5316615	1.9352894	0	0.00627027	0.011
KEGG_OXIDATIVE_PHOSPHORYLATION	109	0.45912483	1.842193	0	0.02433797	0.06
KEGG_PARKINSONS_DISEASE	108	0.4482542	1.8021054	0	0.03072979	0.099
KEGG_FRUCTOSE_AND_MANNOSE_METABOLISM	32	0.53406686	1.7226743	0.00380952	0.05994333	0.224
KEGG_BUTANOATE_METABOLISM	33	0.5276093	1.6830931	0.0094697	0.07439231	0.319
KEGG_CYTOSOLIC_DNA_SENSING_PATHWAY	43	0.44892675	1.5230733	0.02480916	0.2722843	0.803
KEGG_GRAFT_VERSUS_HOST_DISEASE	18	0.54067326	1.5114006	0.03944773	0.26209706	0.835
KEGG_STEROID_BIOSYNTHESIS	17	0.5389776	1.5104612	0.05343511	0.23531026	0.838
KEGG_GLYCOLYSIS_GLUCONEOGENESIS	56	0.4155577	1.5070602	0.01541426	0.21801564	0.846
KEGG_CYTOKINE_CYTOKINE_RECEPTOR_INTERACTION	197	0.34570226	1.502887	0.0017762	0.20495717	0.857
KEGG_HUNTINGTONS_DISEASE	164	0.35330614	1.5002633	0.00170068	0.19295803	0.868
KEGG_AMINO_SUGAR_AND_NUCLEOTIDE_SUGAR_METABOLISM	42	0.4381724	1.4954516	0.0310219	0.18418214	0.873
KEGG_GALACTOSE_METABOLISM	24	0.49400884	1.493101	0.03180914	0.17509758	0.877
KEGG_METABOLISM_OF_XENOBIOTICS_BY_CYTOCHROME_P450	50	0.4093204	1.453237	0.0323741	0.22599593	0.95
KEGG_BLADDER_CANCER	41	0.42557076	1.4399163	0.04323308	0.23295492	0.961
KEGG_VALINE_LEUCINE_AND_ISOLEUCINE_DEGRADATION	44	0.41345498	1.4374782	0.03551402	0.22274841	0.962
KEGG_FATTY_ACID_METABOLISM	41	0.42373234	1.4245584	0.04036697	0.23309301	0.976
KEGG_TYROSINE_METABOLISM	34	0.43931115	1.4240425	0.05323194	0.22209801	0.976
KEGG_ALZHEIMERS_DISEASE	147	0.33042386	1.4001417	0.01094891	0.25217772	0.985
KEGG_P53_SIGNALING_PATHWAY	64	0.37650755	1.3924896	0.03690037	0.25440663	0.988
KEGG_COMPLEMENT_AND_COAGULATION_CASCADES	57	0.38298565	1.3846358	0.04752852	0.25584307	0.991
KEGG_GAP_JUNCTION	83	0.35940734	1.3708907	0.02573529	0.2696298	0.995
KEGG_PRIMARY_IMMUNODEFICIENCY	24	0.44865113	1.357959	0.07339449	0.28243303	0.998
KEGG_STEROID_HORMONE_BIOSYNTHESIS	34	0.41790196	1.3535304	0.08914729	0.27886382	0.998
KEGG_LYSOSOME	116	0.3170462	1.28857	0.07191781	0.41091797	1
KEGG_CHEMOKINE_SIGNALING_PATHWAY	164	0.30074504	1.2801428	0.03723404	0.41790593	1

Mentioned in the Text

KEGG: AS Deletion Neurons

DOWN

NAME	SIZE	ES	NES	NOM p-val	FDR q-val	FWER p-val
KEGG_LONG_TERM_DEPRESSION	62	-0.4619342	-1.6945065	0	0.4060894	0.284
KEGG_ALZHEIMERS_DISEASE	145	-0.3698002	-1.5450963	0.0021322	0.82666373	0.747
KEGG_DRUG_METABOLISM_OTHER_ENZYMES	38	-0.4680263	-1.5383085	0.02282158	0.5832817	0.764
KEGG_SMALL_CELL_LUNG_CANCER	83	-0.396	-1.5047491	0.0087146	0.5771273	0.856
KEGG_CALCIUM_SIGNALING_PATHWAY	159	-0.3523216	-1.4686618	0.01434426	0.6066712	0.922
KEGG_LYSINE_DEGRADATION	44	-0.4264061	-1.4446168	0.02589642	0.60743296	0.953
KEGG_PARKINSONS_DISEASE	108	-0.357089	-1.4245723	0.01026694	0.6023005	0.973
KEGG_PRION_DISEASES	31	-0.4528733	-1.4236437	0.06746032	0.53044367	0.973
KEGG_VASCULAR_SMOOTH_MUSCLE_CONTRACTION	102	-0.3595672	-1.4170115	0.01207244	0.49426493	0.978
KEGG_MELANOGENESIS	92	-0.3574246	-1.397897	0.03067485	0.5071475	0.985
KEGG_ALANINE_ASPARTATE_AND_GLUTAMATE_METABOLISM	30	-0.4466879	-1.3961729	0.07489879	0.46770537	0.986
KEGG_ECM_RECEPTOR_INTERACTION	79	-0.365923	-1.3891255	0.03151261	0.45221663	0.987
KEGG_SELENOAMINO_ACID_METABOLISM	24	-0.4673972	-1.373588	0.08033827	0.46872815	0.99
KEGG_LONG_TERM_POTENTIATION	62	-0.3726961	-1.3586869	0.04990403	0.4833538	0.993
KEGG_GLIOMA	62	-0.3725277	-1.3579254	0.0491453	0.4530296	0.993
KEGG_AMYOTROPHIC_LATERAL_SCLEROSIS_ALS	46	-0.392426	-1.3503655	0.06378601	0.44834524	0.995
KEGG_PANTOTHENATE_AND_COA_BIOSYNTHESIS	15	-0.5100718	-1.3433353	0.14609054	0.44290158	0.997
KEGG_SPHINGOLIPID_METABOLISM	35	-0.4174554	-1.3421384	0.09696969	0.42120445	0.997
KEGG_RENIN_ANGIOTENSIN_SYSTEM	15	-0.4954344	-1.3377883	0.11195446	0.41043818	0.998
KEGG_NOTCH_SIGNALING_PATHWAY	43	-0.3982469	-1.3349452	0.0724346	0.39750975	0.998
KEGG_VIBRIO_CHOLERAE_INFECTION	53	-0.3761694	-1.3150212	0.10240964	0.4308302	1

UP

NAME	SIZE	ES	NES	NOM p-val	FDR q-val	FWER p-val
KEGG_STEROID_BIOSYNTHESIS	17	0.79428303	2.1545434	0	7.91E-04	0.001
KEGG_FRUCTOSE_AND_MANNOSE_METABOLISM	33	0.60110503	1.8836194	0.00192308	0.01389321	0.037
KEGG_STEROID_HORMONE_BIOSYNTHESIS	40	0.54847443	1.8275898	0	0.01820514	0.071
KEGG_TERPENOID_BACKBONE_BIOSYNTHESIS	15	0.6516438	1.7261577	0.01145038	0.04510398	0.21
KEGG_PENTOSE_PHOSPHATE_PATHWAY	25	0.5772126	1.683968	0.0113852	0.05559753	0.301
KEGG_GLYCOLYSIS_GLUCONEOGENESIS	58	0.45955414	1.6402068	0.00393701	0.07311387	0.438
KEGG_METABOLISM_OF_XENOBIOTICS_BY_CYTOCHROME_P45(	57	0.4583219	1.6067728	0.00401606	0.08702132	0.559
KEGG_RNA_POLYMERASE	29	0.5114668	1.5849962	0.00578035	0.09407733	0.637
KEGG_BASAL_TRANSCRIPTION_FACTORS	33	0.48395783	1.5266265	0.03428571	0.13768664	0.802
KEGG_BIOSYNTHESIS_OF_UNSATURATED_FATTY_ACIDS	22	0.46971262	1.3734919	0.09942639	0.3916363	0.997
KEGG_RIBOSOME	86	0.35412627	1.372683	0.0368217	0.35812816	0.997
KEGG_ALDOSTERONE_REGULATED_SODIUM_REABSORPTION	37	0.41842628	1.3668619	0.07677165	0.34231362	0.998
KEGG_PPAR_SIGNALING_PATHWAY	62	0.3655422	1.3473259	0.06262231	0.3612048	0.999
KEGG_GLYCEROLIPID_METABOLISM	47	0.38226116	1.3127617	0.06967213	0.42007983	1
KEGG_AMINO_SUGAR_AND_NUCLEOTIDE_SUGAR_METABOLISN	42	0.38038158	1.2725906	0.10836502	0.5034263	1
KEGG_GLYCOSAMINOGLYCAN_DEGRADATION	21	0.40839535	1.1981012	0.21301775	0.73112905	1
KEGG_GLYCOSAMINOGLYCAN_BIOSYNTHESIS_CHONDROITIN_SU	20	0.42574117	1.1892812	0.2267658	0.7216315	1
KEGG_STARCH_AND_SUCROSE_METABOLISM	40	0.353763	1.1885904	0.18958333	0.6851217	1
KEGG_THYROID_CANCER	28	0.38601628	1.1878542	0.21359223	0.65200734	1
KEGG_PROTEASOME	41	0.35376793	1.1820115	0.20292504	0.63990986	1

Mentioned in the Text

Also of interest
